# Targeting CTP synthetase 1 to restore interferon induction and impede nucleotide synthesis in SARS-CoV-2 infection

**DOI:** 10.1128/mbio.00649-25

**Published:** 2025-04-29

**Authors:** Youliang Rao, Chao Qin, Bianca Espinosa, Ting-Yu Wang, Shu Feng, Ali Can Savas, Jill Henley, Lucio Comai, Chao Zhang, Pinghui Feng

**Affiliations:** 1 Section of Infection and Immunity, Herman Ostrow School of Dentistry, University of Southern California, Los Angeles, California, USA; 2 Department of Chemistry, Dornsife College of Arts, Letters and Sciences, University of Southern California, Los Angeles, California, USA; 3 Department of Molecular Microbiology and Immunology, Keck School of Medicine, University of Southern California, Los Angeles, California, USA; The University of North Carolina at Chapel Hill, Chapel Hill, North Carolina, USA

**Keywords:** SARS-CoV-2, interferon, pyrimidine metabolism, CTPS1, antiviral pharmacology

## Abstract

Despite the global impact caused by the most recent SARS-CoV-2 pandemic, our knowledge of the molecular underpinnings of its highly infectious nature remains incomplete. We report here that SARS-CoV-2 exploits cellular CTP synthetase 1 (CTPS1) to promote CTP synthesis and suppress interferon (IFN) induction. In addition to catalyzing CTP synthesis, CTPS1 also deamidates interferon regulatory factor 3 (IRF3) to dampen interferon induction. Screening a SARS-CoV-2 expression library, we identified several viral proteins that interact with CTPS1. Functional analyses demonstrate that ORF8 and Nsp8 activate CTPS1 to deamidate IRF3 and negate IFN induction, whereas ORF7b and ORF8 activate CTPS1 to promote CTP synthesis. These results highlight CTPS1 as a signaling node that integrates cellular metabolism and innate immune response. Indeed, small-molecule inhibitors of CTPS1 deplete CTP and boost IFN induction in SARS-CoV-2-infected cells, thus effectively impeding SARS-CoV-2 replication and pathogenesis in mouse models. Our work uncovers an intricate mechanism by which a viral pathogen couples immune evasion to metabolic activation to fuel viral replication. Inhibition of the cellular CTPS1 offers an attractive means to develop antiviral therapy against highly mutagenic viruses.

**IMPORTANCE:**

Our understanding of the underpinnings of highly infectious SARS-CoV-2 is rudimentary at best. We report here that SARS-CoV-2 activates CTPS1 to promote CTP synthesis and suppress IFN induction, thus coupling immune evasion to activated nucleotide synthesis. Inhibition of the key metabolic enzyme not only depletes the nucleotide pool but also boosts host antiviral defense, thereby impeding SARS-CoV-2 replication. Targeting cellular enzymes presents a strategy to counter the rapidly evolving SARS-CoV-2 variants.

## INTRODUCTION

First reported in December 2019, the coronavirus disease known as COVID-19 rapidly spread worldwide and became a global pandemic. The etiological agent of COVID-19 was soon identified as a new coronavirus, severe acute respiratory syndrome coronavirus 2 (SARS-CoV-2) (1, 2). Compared with other zoonotic coronaviruses including SARS-CoV and MERS-CoV, SARS-CoV-2 is highly infectious and transmissible (3, 4). Current efforts have extensively focused on the entry step, which is primarily mediated by the interaction between the SARS-CoV-2 spike (S) protein and the human angiotensin-converting enzyme 2 (hACE2) (1, 5). Structural and functional analyses of this receptor-ligand interaction indicate that the SARS-CoV-2 S protein evolved a higher affinity for binding to hACE2 on target cells, partly explaining the highly infectious nature of SARS-CoV-2 during the COVID-19 pandemic (6). However, the viral mechanisms downstream of viral entry that contribute to the infection and pathogenesis of SARS-CoV-2 are not well understood. The innate immune response constitutes the first line of defense against intracellular pathogens such as viruses (7). To efficiently replicate within an immune-competent host, a virus must overcome the barrier of the host’s innate immune defense, chiefly mediated by the interferon system (8). Indeed, previous studies of SARS-CoV-2 infection involving patient samples, model animals, and cell lines suggest that SARS-CoV-2 either weakly induces IFNs or inhibits IFN induction (9–11).

In addition to overcoming innate immune defense, viruses rely on cellular machinery to synthesize macromolecules and biomaterials that are subsequently assembled into progeny virions (12, 13). Thus, viruses often activate and redirect cellular biosynthetic activities to facilitate the production of viral components, such as proteins, nucleic acids, and lipids, which constitute essential building blocks of virions (14, 15). Central to viral replication is the reprogramming of cellular metabolic processes that are often activated to provide precursors for viral biosynthesis in infected cells (12). The highly infectious nature of SARS-CoV-2 likely involves molecular interactions that boost the rate-limiting steps of key metabolic pathways to fuel viral replication and subsequent dissemination (16, 17). Cellular glutamine amidotransferases (GATs) catalyze the synthesis of nucleotides, amino acids, glycoproteins, and an enzyme cofactor (NAD) (18, 19). Our studies have shown that these enzymes are capable of deamidating key signaling molecules, such as those involved in innate immune defense, to modulate fundamental biological processes (20). For example, CTP synthetase 1 (CTPS1) deamidates IRF3 at N85. Deamidated IRF3 fails to bind the promoters of classic IRF3-responsible genes, thus muting IFN induction (21). Here, we reported that SARS-CoV-2 polypeptides activate CTPS1 to promote *de novo* CTP synthesis and deamidate IRF3, thus coupling immune evasion to activate nucleotide synthesis. As such, several small molecules that were developed as CTPS1 inhibitors restored IFN induction and depleted CTP in SARS-CoV-2-infected cells, thus impeding SARS-CoV-2 replication. This study unravels a viral strategy that hijacks a cellular CTP synthesis enzyme to fuel nucleotide synthesis and shut down IFN induction, forging a molecular link between metabolism and innate immune defense. CTPS1 blockade depletes CTP supply and restores innate immune response, which can yield antiviral therapy that is resistant to SARS-CoV-2 genetic variation.

## RESULTS

### SARS-CoV-2 suppresses IFN induction via inducing IRF3 deamidation

**Fig 1:**
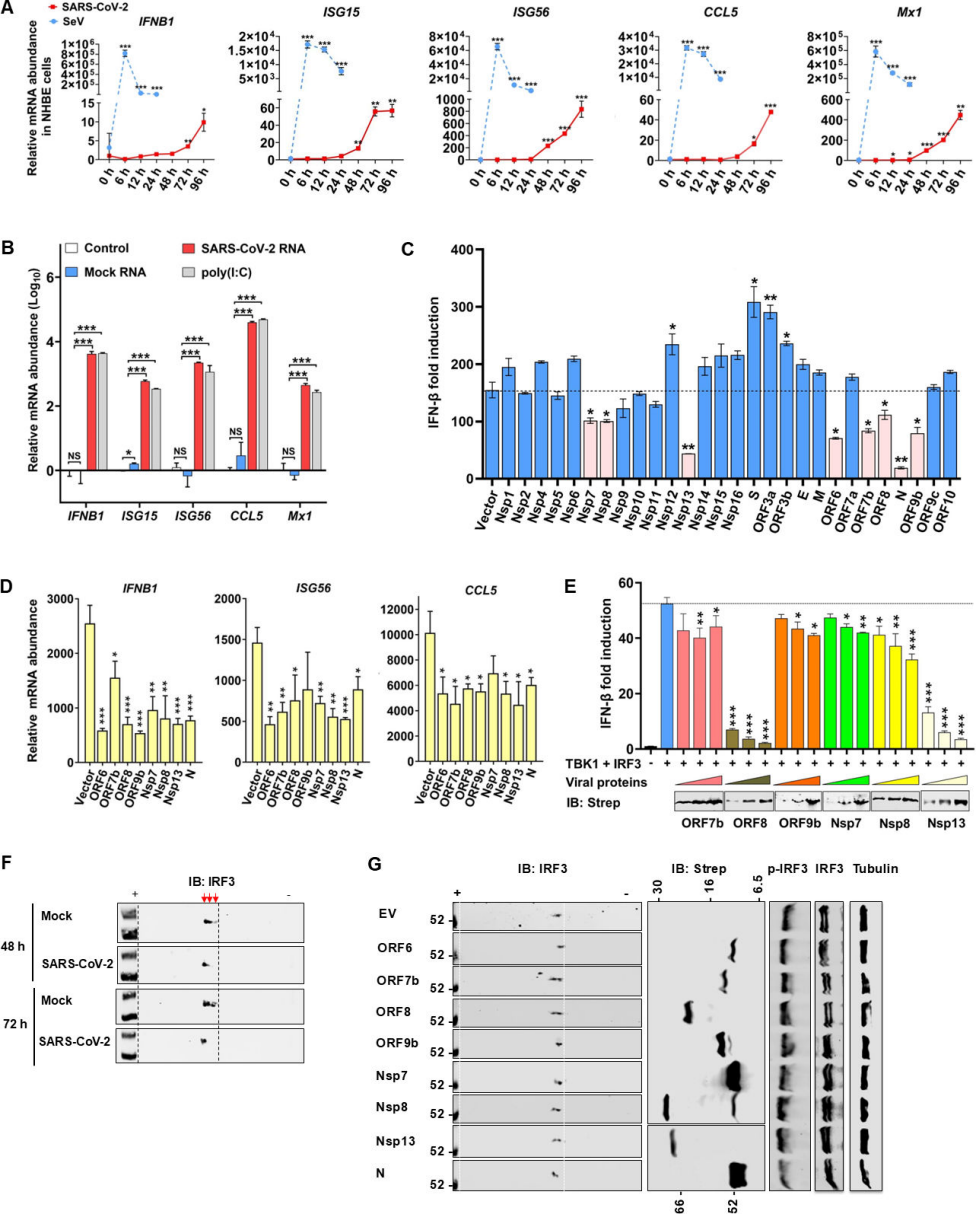
SARS-CoV-2 inhibits IFN induction via inducing IRF3 deamidation. (**A**) Normal human bronchial epithelial (NHBE) cells were infected with Sendai virus (SeV) (100 HAU/mL) or SARS-CoV-2 (MOI = 1). Total RNA was extracted, reverse-transcribed, and analyzed by real-time PCR with primers specific for *IFNB1*, *ISG15*, *ISG56*, *CCL5,* and *Mx1*. (**B**) NHBE cells were transfected with poly(I:C) and RNA purified from the medium of Vero E6-hACE2 cells infected with or without SARS-CoV-2 (72 hpi). The mRNA abundance of immune genes was analyzed by real-time PCR at 6 h post-transfection. (**C**) Modulation of IFN-β induction was determined by promoter activity in 293T cells expressing indicated SARS-CoV-2 proteins, with SeV infection. (**D**) Inhibition of antiviral gene expression by SARS-CoV-2 proteins in 293T cells infected with SeV was examined by real-time PCR with primers specific for indicated genes. (**E**) Inhibition of IFN-β induction by selected SARS-CoV-2 proteins was determined by reporter assay of 293T cells expressing TBK1 and IRF3. (**F**) Caco-2 cells were infected with SARS-CoV-2 for 48 h and 72 h with MOI 0.5. IRF3 charge status was analyzed by two-dimensional gel electrophoresis and immunoblotting. (**G**) Effects of selected SARS-CoV-2 proteins on IRF3 charge status and phosphorylation were determined by two-dimensional gel electrophoresis and immunoblotting analyses using lysates of 293T cells transfected with plasmids containing indicated genes. Data are presented as means ± SD of biological triplicates (**A–E**) and are representative of three independent experiments (**F and G**). Statistical significance was calculated using a one-way ANOVA test or unpaired, two-tailed Student’s *t*-test. *P < 0.05; **P < 0.01; ***P < 0.001.

SARS-CoV-2 is highly infectious and transmissible in the human population. We hypothesized that SARS-CoV-2 encodes a number of viral polypeptides to modulate host immune response and metabolism, thereby promoting viral replication and dissemination. To test this hypothesis, we first compared the antiviral gene expression induced by SARS-CoV-2 with that induced by Sendai virus, a prototype RNA virus. In normal human bronchial epithelial cells (NHBE), the Sendai virus triggered a rapid and robust expression of multiple antiviral genes with a peak at 6 h post-infection (hpi) with the fold of increase ranging from ~15,000 (for *ISG15*) to 800,000 (for *IFNB1*) (Fig. 1A). By stark contrast, SARS-CoV-2 induced a weak and delayed expression of antiviral genes (Fig. 1A). The fold of induction of these antiviral genes by SeV was roughly three orders of magnitude higher than that induced by SARS-CoV-2. Similar patterns were observed in human Calu-3 lung cancer cells and Caco-2 colorectal cancer cells, two model cell lines that support robust SARS-CoV-2 replication (Fig. S1A and B). Interestingly, although delayed, the expression of *Mx1* in Calu-3 and Caco-2 cells induced by SARS-CoV-2 was as robust as that induced by SeV. To determine whether RNA derived from SARS-CoV-2-infected cells is able to provoke innate immune activation, we extracted total RNA from SARS-CoV-2-infected NHBE cells and transfected it, along with poly(I:C), into NHBE cells. When antiviral gene expression was profiled, we found that the total RNA extracted from SARS-CoV-2-infected NHBE cells, but not that from mock-infected NHBE cells, induced antiviral gene expression as potently as poly(I:C) (Fig. S1C). To eliminate the side effect induced by cellular mRNA on antiviral gene expression, we transfected NHBE cells with vRNA purified from the medium of SARS-CoV-2-infected Vero E6-hACE2 cells. Compared with RNA purified from the medium of mock-infected Vero E6-hACE2 cells, vRNA induced more potent expression of antiviral genes, which is even stronger than that induced by poly(I:C) (Fig. 1B). These results support the conclusion that SARS-CoV-2 suppresses antiviral innate immune defense.

To understand the viral mechanisms of immune modulation, we screened a SARS-CoV-2 expression library for inhibitors of IFN-β induction by Sendai virus (22). This reporter assay identified several SARS-CoV-2 proteins, including ORF6, ORF7b, ORF8, ORF9b, N, Nsp7, Nsp8, and Nsp13, capable of negating IFN induction to various extents (Fig. 1C). Indeed, when transiently expressed in 293T cells, all eight viral polypeptides inhibited the expression of antiviral genes, including IFNB1, ISG56, and CCL5, upon SeV infection (Fig. 1D). Importantly, ORF6 and N were previously reported to inhibit the nuclear import of transcription factors (e.g., IRF3) and sequester viral double-stranded RNA, respectively (23, 24), to suppress innate immune defense. Thus, we further examined the other six viral proteins for the inhibition of IFN induction. Nevertheless, these results demonstrate that multiple viral polypeptides can inhibit IFN induction.

SARS-CoV-2 is a coronavirus that likely induces innate immune activation via RNA sensors such as RIG-I and MDA5. To determine the target of inhibition, we over-expressed key components of this pathway, including RIG-I-N (2CARD-only), MAVS, TBK1, and IRF3, and examined IFN-β induction by reporter assay (Fig. S1D). Notably, RIG-I-N is a constitutively active form of RIG-I independent of RNA ligand. This assay showed that ORF7b, ORF8, and Nsp13 could significantly inhibit IFN induction by the expression of more than one component of the RIG-I-IFN pathway, whereas ORF9b, Nsp7, and Nsp8 did not significantly inhibit IFN induction by ectopic expression (Fig. S1E). Further analysis demonstrated that ORF8 and Nsp13 potently inhibited IFN induction by over-expressing IRF3 and TBK1, suggesting that IRF3 is the point of inhibition (Fig. 1E). Thus, we focused on IRF3 regulation by these viral polypeptides, with a keen interest in deamidation that can be catalyzed by CTPS1 (21). When endogenous IRF3 was analyzed by two-dimensional gel electrophoresis (2-DGE), we found that SARS-CoV-2 infection shifted IRF3 toward the positive end of the gel strips, consistent with the outcome of deamidation (Fig. 1F). Further analysis of viral polypeptides revealed that ORF7b, ORF8, Nsp8, and Nsp13 induced a shift of IRF3 toward the positive end of the gel strip, suggesting the deamidation of IRF3 (Fig. 1G). However, these viral proteins did not alter IRF3 phosphorylation, another well-characterized modification that reduces protein charge (Fig. 1G). These results collectively show that SARS-CoV-2 targets IRF3 for inhibition.

### SARS-CoV-2 promotes CTPS1-mediated IRF3 deamidation to impede IFN induction

**Fig 2:**
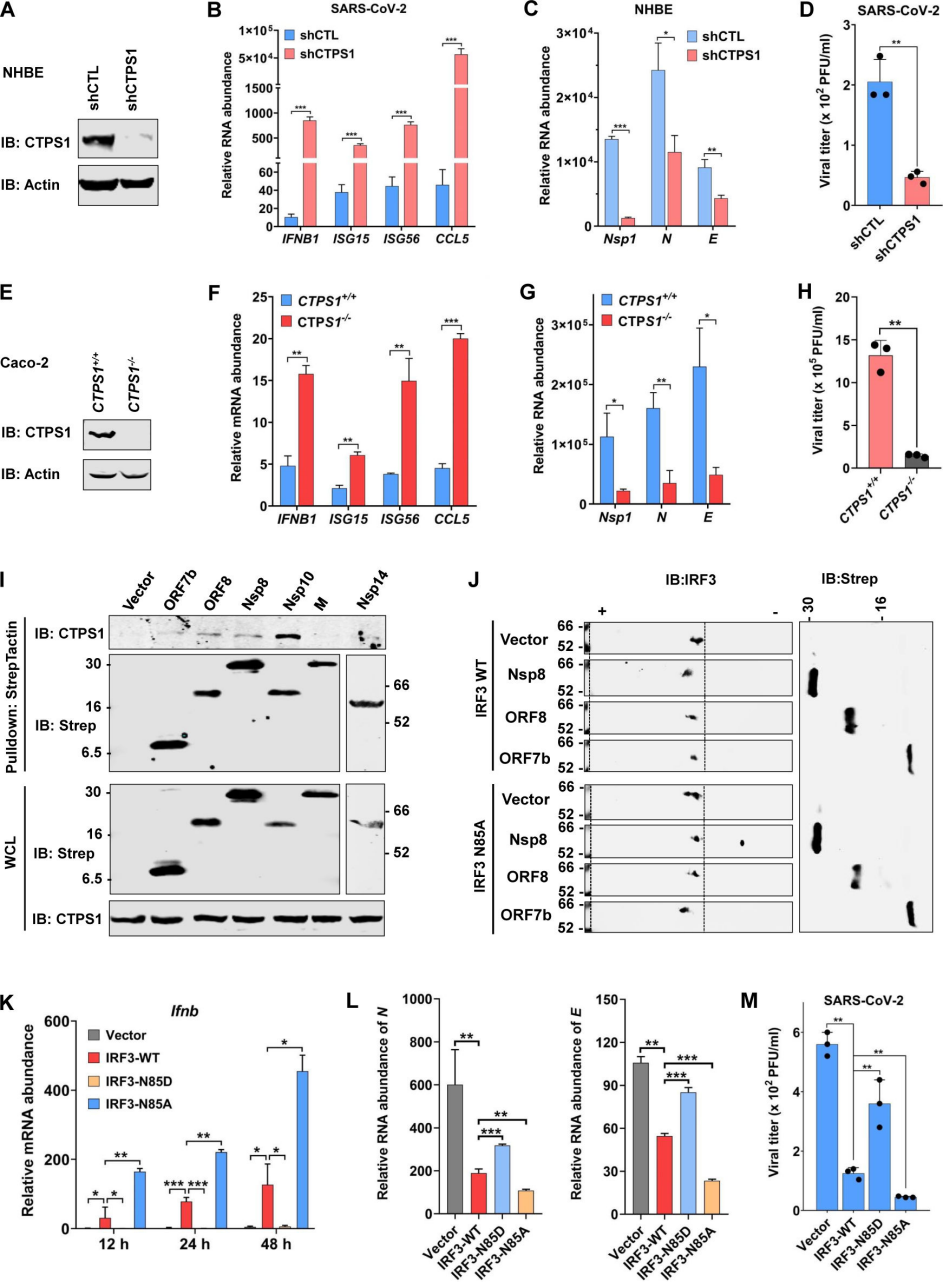
CTPS1 negatively regulates SARS-CoV-2-mediated IFN induction. (**A**) CTPS1 depletion in NHBE cells was determined by immunoblotting. (**B through D**) Effects of CTPS1 depletion on the expression of cellular antiviral genes (**B**) and viral genes (**C**) were determined by real-time PCR analysis of total RNA extracted at 48 h after SARS-CoV-2 infection (MOI = 0.1). Medium of NHBE cells infected with SARS-CoV-2 was used for plaque assay to determine infectious viral progeny (**D**). (**E**) Knockout of *CTPS1* in Caco-2 cells was analyzed by immunoblotting using cells from individual clones. (**F through H**) *CTPS1^−/−^* Caco-2 cells were infected with SARS-CoV-2 for 24 h with MOI 0.5 (***F***) or 72 h with MOI 0.1 (**G and H**). Antiviral gene expression and viral RNA abundance in the cells were determined by real-time PCR (**F and G**). Viral titer in the medium was analyzed by plaque assay in Vero E6-hACE2 cells (**H**). (**I**) Interactions between endogenous CTPS1 and SARS-CoV-2 proteins were analyzed by co-immunoprecipitation in transfected 293T cells. Strep is a tag for SARS-CoV-2 proteins. (**J**) The effect of SARS-CoV-2 proteins on the charge status of IRF3-WT and IRF3-N85A was determined by two-dimensional gel electrophoresis and immunoblotting in wild-type and IRF3-N85A knock-in 293T cells. (**K through M**) Human ACE2-expressing *Irf3^−/−^Irf7^−/−^* MEFs were reconstituted with IRF3-WT, IRF3-N85D, IRF3-N85A, and vector. The effect of IRF3 and its mutants on *Ifnb* expression (**K**) and SARS-CoV-2 RNA abundance (**L**) was assessed by real-time PCR with total RNA extracted at 24 h after SARS-CoV-2 infection (MOI = 0.01). Medium of SARS-CoV-2-infected MEFs was used for plaque assay to determine infectious viral progeny (**M**). Data are presented as means ± SD of biological triplicates (**B through D, F through H, K through M**) and are representative of three independent experiments (A, E, I, and J). Statistical significance was calculated using the two-way ANOVA test, one-way ANOVA test, or unpaired, two-tailed Student’s *t*-test. *P < 0.05; **P < 0.01; ***P < 0.001.

We recently reported that CTPS1 deamidates IRF3 at N85 to negate IFN induction (21). To assess the role of CTPS1 in SARS-CoV-2 infection, we first depleted CTPS1 in NHBE cells (Fig. 2A) and assessed antiviral gene expression. Real-time PCR analysis indicated that depletion of CTPS1 increased the expression of *IFNB1*, *ISG15*, *ISG56*, and *CCL5* by a factor ranging from ~15 (for *ISG15* and *ISG56*) to 1,000 (for *CCL5*) (Fig. 2B). Conversely, depletion of CTPS1 reduced SARS-CoV-2 RNA abundance by 2-fold for *N* and *E* genes and >10-fold for Nsp1 gene (Fig. 2C), which correlated with a 4-fold reduction in viral titer in the medium at 48 h post-infection (Fig. 2D). The peak timing of viral gene expression and progeny virus production differs, leading to variations in fold differences. Similar results were observed in Caco-2 cells for elevated antiviral gene expression in response to SARS-CoV-2 infection upon CTPS1 depletion (Fig. S2A and B). This result also correlated with reduced SARS-CoV-2 replication as analyzed by real-time PCR for viral RNA abundance and plaque assay for infectious virions in the medium (Fig. S2C and D). Consistently, knockout of CTPS1 in Caco-2 cells potently elevated antiviral gene expression and inhibited viral RNA abundance and viral titer in response to SARS-CoV-2 infection (Fig. 2E through H). These results show that CTPS1 negatively regulates antiviral immune response against SARS-CoV-2 and that deficiency in CTPS1 promotes antiviral gene expression to impede SARS-CoV-2 replication.

To probe the virus-host interaction underpinning CTPS1-mediated IRF3 deamidation, we screened for viral proteins that interact with CTPS1 using a SARS-CoV-2 expression library (22). A co-IP assay identified multiple SARS-CoV-2 polypeptides that co-precipitated with CTPS1 in transfected 293T cells, including ORF7b, ORF8, M, Nsp8, Nsp10, and Nsp14 (Fig. S2E). The interaction between CTPS1 and these SARS-CoV-2 proteins, except M, was further validated by co-IP assay using endogenous CTPS1 (Fig. 2I). Three out of the six CTPS1-interacting SARS-CoV-2 polypeptides, that is, ORF7b, ORF8, and Nsp8, also induced IRF3 deamidation in transfected 293T cells (Fig. 1G). We reasoned that these SARS-CoV-2 polypeptides usurp CTPS1 to promote IRF3 deamidation. To test this, we expressed ORF7b, ORF8, and Nsp8 in wild-type, IRF3-N85A, and IRF3-N85D knock-in 293T cells for two-dimensional gel electrophoresis. This experiment revealed that wild-type IRF3, but not the IRF3-N85A and IRF3-N85D mutants, was shifted by ORF8 and Nsp8 expressions (Fig. 2J; Fig. S2F). Interestingly, ORF7b expression shifted wild-type IRF3 and IRF3-N85A, suggesting that ORF7b likely induces the deamidation of IRF3 at sites other than N85. Nevertheless, these results show that ORF8 and Nsp8 induce the CTPS1-mediated deamidation of IRF3 at N85.

To determine the effect of IRF3 deamidation on SARS-CoV-2 replication, we established *Irf3^−/−^Irf7^−/−^* MEFs stably expressing human ACE2 to facilitate SARS-CoV-2 infection. Then, the hACE2-expressed *Irf3^−/−^Irf7^−/−^* MEFs were reconstituted with wild-type IRF3, IRF3-N85D, and IRF3-N85A (Fig. S2G). These “reconstituted” MEFs were infected with SARS-CoV-2 and examined for innate immune response. Quantitative real-time analyses indicated that wild-type IRF3 induced a modest level of expression of *Ifnb*, *Isg15*, *Isg56,* and *Cxcl10* (Fig. 2K; Fig. S2H through J). However, IRF3-N85A robustly and IRF3-N85D minimally induced the expression of these genes (Fig. 2K; Fig. S2H through J). Conversely, wild-type IRF3 and IRF3-N85A reduced viral RNAs by ~40% to 70%, whereas IRF3-N85D had no apparent effect on the RNA levels of *Nsp1* and *E* or reduced *N* RNA by ~45% (Fig. 2L; Fig. S2K). Plaque assay further showed that wild-type IRF3 and IRF3-N85A diminished infectious SARS-CoV-2 in the medium by 75% and 90%, respectively, whereas IRF3-N85D reduced by ~30% (Fig. 2M). Thus, deamidation of N85 inactivates IRF3 in host defense against SARS-CoV-2 infection.

### SARS-CoV-2 polypeptides increase CTPS1 enzymatic activities

**Fig 3:**
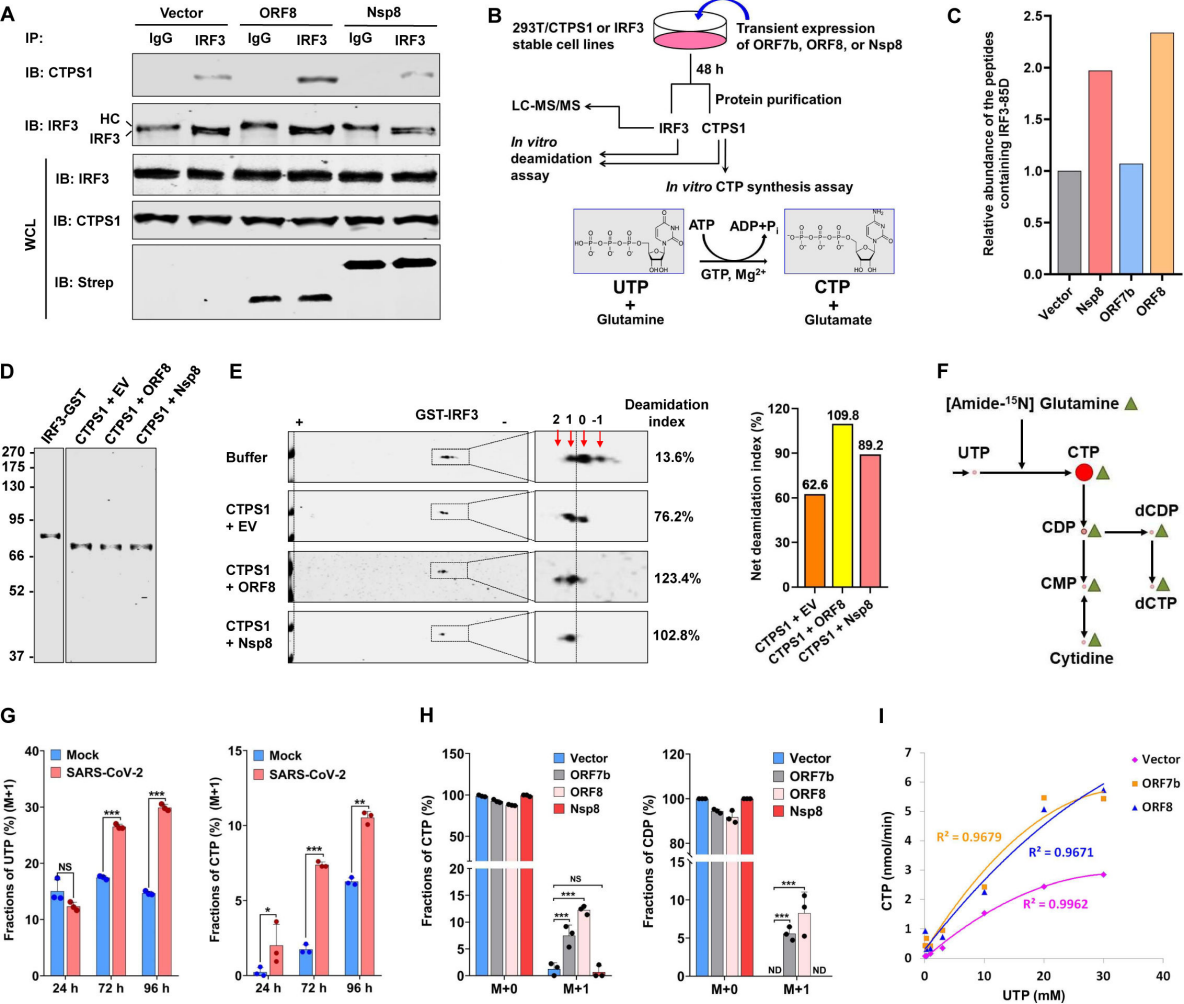
SARS-CoV-2 polypeptides increase CTPS1 enzymatic activities. (**A**) The effect of SARS-CoV-2 ORF8 and Nsp8 on the interaction between endogenous CTPS1 and IRF3, in 293T cells expressing ORF8 and Nsp8, was analyzed by co-immunoprecipitation and immunoblotting. (**B**) Schematic diagram of LC-MS/MS, *in vitro* deamidation assay, and *in vitro* CTPS1 enzymatic assay, with expression of SARS-CoV-2 proteins. (**C**) LC-MS/MS analysis was performed with purified FLAG-IRF3 from stable 293T cells expressing ORF7b, ORF8, and Nsp8, as described in panel **B**. Deamidated IRF3-85N induced by viral proteins was normalized with IRF3-85D abundance in the vector group. This data were obtained from one or two independent repeat experiments. (**D**) IRF3-GST was purified from 293T cells, and FLAG-CTPS1 was purified from 293T/FLAG-CTPS1 stable cells transiently transfected with a plasmid expressing SARS-CoV-2 ORF8 or Nsp8. Purified proteins were analyzed by Coomassie blue staining. (**E**) Left panel: *in vitro* IRF3 deamidation was performed with CTPS1 purified from stable 293T cells with ORF8 and Nsp8 expression, as described in panel **B**. The reactions were analyzed by two-dimensional gel electrophoresis and immunoblotting. Numbers indicated deamidation events. The deamidation index is the composite of deamidated percentage (intensity of the species) × deamidation events (number). For the buffer group, the deamidation index is obtained by 24.8% × 1 + 11.2% × (−1) = 13.6%, which is the basal deamidation level. Deamidation index from CTPS1 with ORF8 is obtained by 24.5% × 2 + 74.4% × 1 = 123.4%. Right panel: net deamidation index is obtained by subtracting the basal deamidation level from the total deamidation index. These data were obtained from one or two independent experiments. (**F**) Diagram of nitrogen incorporation in CTP synthesis using [amide-^15^N]glutamine. (**G**) Intracellular UTP and CTP labeled with ^15^N were analyzed at 24, 72, and 96 h after SARS-CoV-2 (MOI = 1) infection by mass spectrometry. Metabolites with M + 2 are below the detection limit. (**H**) The effects of SARS-CoV-2 proteins on intracellular CTP and CDP labeled with ^15^N were determined by mass spectrometry in Caco-2 cells infected with lentivirus carrying SARS-CoV-2 ORF7b, ORF8, Nsp8, and control vector. M + a indicates targeted metabolites labeled with [amide-^15^N] of glutamine. Metabolites with M + 2 are below the detection limit. ND, not detected. (**I**) Effect of SARS-CoV-2 ORF7b and ORF8 on CTPS1 activity in CTP synthesis, in the presence of 2 mM ATP, 2 mM L-glutamine, 0.1 mM GTP, and increasing concentrations of UTP, was determined by *in vitro* enzymatic assay as described in panel **B** and analyzed by mass spectrometry. Data are presented as means ± SD of biological triplicates (**G and H**) and are representative of three independent experiments (A, D, and E). Statistical significance was calculated using the one-way ANOVA test. *P < 0.05; **P < 0.01; ***P < 0.001.

To dissect the mechanism by which SARS-CoV-2 polypeptides promote CTPS1-mediated deamidation of IRF3, we determined whether ORF8 and Nsp8 impact the CTPS1-IRF3 interaction by co-IP assays. In 293T cells transiently expressing ORF8, more CTPS1 was precipitated by IRF3, indicating an elevated interaction between CTPS1 and IRF3 (Fig. 3A). By contrast, Nsp8 had no apparent effect on this interaction. Next, we determined whether ORF8 and Nsp8 affect the IRF3-deamidating activity of CTPS1. To do that, we purified IRF3 from 293T cells expressing Nsp8, ORF7b, and ORF8 for LC-MS/MS analysis. Compared with the control group (vector), both Nsp8 and ORF8 increased the relative peptide abundance containing 85D (Fig. 3B and C). However, ORF7b had a marginal effect on the relative abundance of the 85D-containing peptide. We then purified CTPS1, with or without SARS-CoV-2 polypeptides, from transfected 293T cells and performed *in vitro* deamidation assay (Fig. 3B and D). Two-dimensional gel electrophoresis analysis indicated that ORF8 and, to a lesser extent, Nsp8 increased CTPS1 activity to deamidate IRF3 (Fig. 3E). Together, these results collectively show that SARS-CoV-2 polypeptides can enhance the IRF3-deamidating activity of CTPS1.

CTPS1 is responsible for the synthesis of CTP, which is crucial for a balanced nucleotide pool during cell proliferation and viral replication. Activated nucleotide synthesis is likely to favor the transcription and genome replication of SARS-CoV-2. We then examined the metabolites of the glycolysis and nucleotide synthesis pathways. In the colorectal Caco-2 cell line that supports SARS-CoV-2 replication, we found that SARS-CoV-2 infection had no significant effect on the intracellular concentration of CTP (Fig. S3A). However, the relative concentrations of UTP, UDP, and, to a lesser extent, UMP significantly increased in Caco-2 cells at 72 h after SARS-CoV-2 infection (Fig. S3B). Strikingly, CTP and UTP significantly decreased at 96 h post-infection. These results support the rate-limiting role of CTP synthetases in catalyzing UTP to CTP conversion and suggest that the decrease of CTP and UTP at 96 hpi is likely due to rapid consumption. To determine the rate of synthesis that reflects the activity of CTPS1, we analyzed CTP synthesis during SARS-CoV-2 infection using isotope tracing with [amide-^15^N]glutamine (Fig. 3F). Compared with mock-infected cells, SARS-CoV-2 increased the labeled CTP (M + 1) by >2-fold at 24 hpi, >3-fold at 72 hpi, and 0.5-fold at 96 hpi in Caco-2 cells (Fig. 3G). Interestingly, SARS-CoV-2 infection had no apparent effect on the [amide-^15^N]UTP (M + 1) at 24 hpi and modestly increased [amide-^15^N]UTP (M + 1) at 72 and 96 hpi under similar conditions, indicating the specificity of CTPS1 activation during SARS-CoV-2 infection. [amide-^15^N]UTP (M + 1) is a product of the *de novo* pyrimidine synthesis where CAD catalyzes dihydroorotate synthesis using glutamine, suggesting an increase in the *de novo* pyrimidine synthesis at 72 and 96 hpi (25). Notably, SARS-CoV-2 infection did not increase CTPS1 protein levels, suggesting CTPS1 activation during viral infection (Fig. S3C). Next, we established Caco-2 cell lines that stably express SARS-CoV-2 polypeptides, including ORF7b, ORF8, and Nsp8, all of which induced IRF3 deamidation in Caco-2 cells (Fig. S3D). When flux analysis with [amide-^15^N]glutamine was performed, we found that cells expressing ORF7b and ORF8 had >3-fold and 5-fold more [amide-^15^N]CTP (M + 1) compared with control cells (Vector group), respectively (Fig. 3H). Consistently, ORF7b and ORF8 also increased [amide-^15^N]CDP (M + 1), an immediate product hydrolyzed from CTP. Strikingly, Nsp8 expression had no apparent effect on labeled [amide-^15^N]CTP. A robust increase in [amide-^15^N]CTP was also observed in ORF8-expressing LoVo colorectal cells (Fig. S3E). ORF8 expression in Caco-2 cells significantly increased cell proliferation, whereas ORF7b slightly increased it, consistent with their role in promoting CTP synthesis (Fig. S3F). These results show that ORF8 and, to a lesser extent, ORF7b promote CTP synthesis.

To probe the effect of ORF7b and ORF8 on the enzymatic activity of CTPS1, we purified CTPS1 from stable 293T/FLAG-CTPS1 cells with transient expression of ORF7b and ORF8 and performed biochemical assays to determine the kinetic parameters, that is, *K*_cat_ and *K*_*m*_, of CTPS1 (Fig. 3B). Compared with the control group (vector), ORF7b and ORF8 expressions increased *K*_cat_ of CTPS1 by ~1-fold and 2-fold, respectively (Fig. 3I; Fig. S3G). Interestingly, ORF8, but not ORF7b, increased *K*_*m*_ of CTPS1 by ~0.7-fold. These results demonstrate that SARS-CoV-2 ORF7b and ORF8 activate CTPS1 to synthesize CTP.

### CTPS1 inhibitors demonstrate antiviral activity against SARS-CoV-2

**Fig 4:**
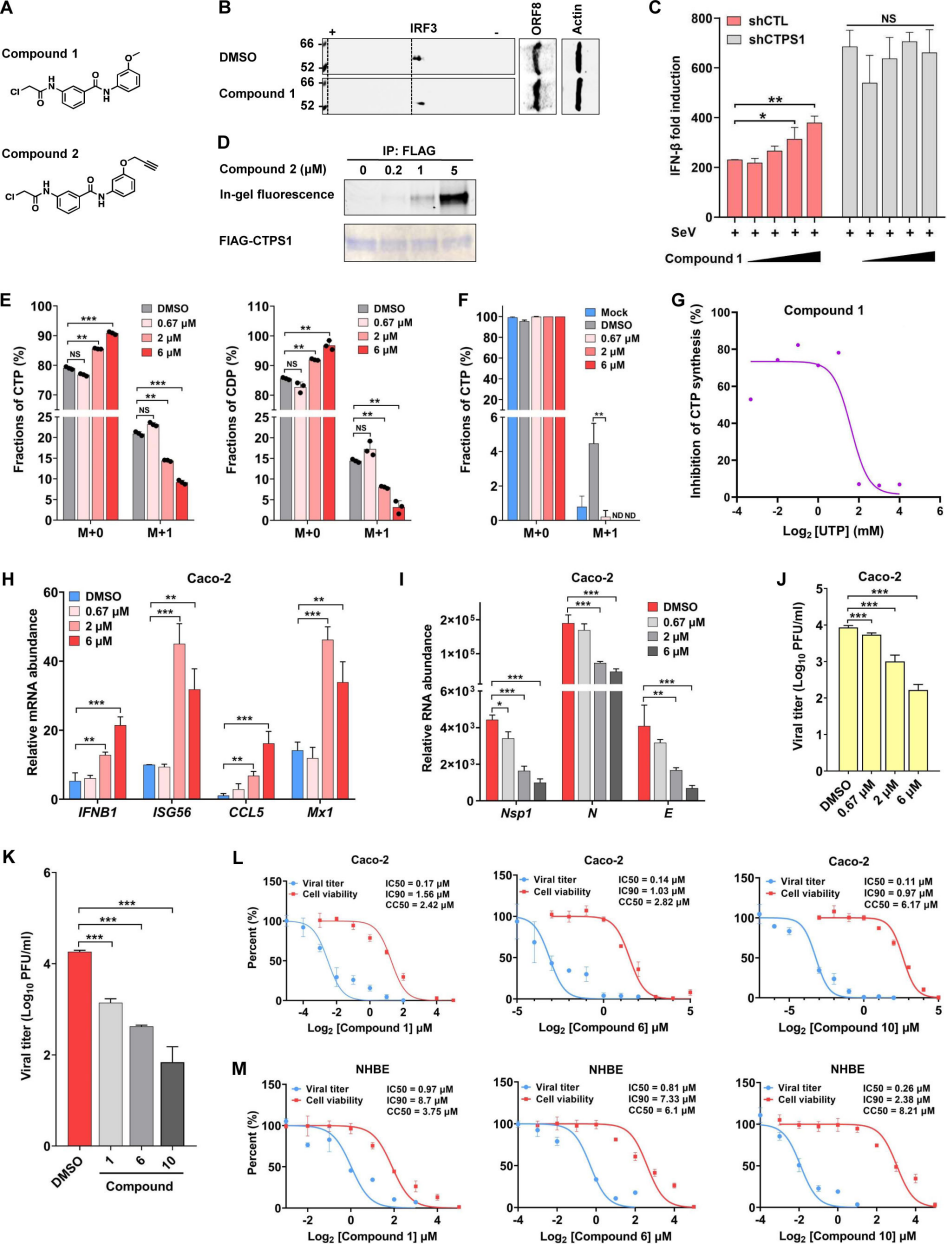
CTPS1 inhibitors impede SARS-CoV-2 replication. (**A**) Structures of compounds 1 and 2. (**B**) Effect of compound 1 on SARS-CoV-2 ORF8-induced IRF3 deamidation was analyzed by two-dimensional gel electrophoresis and immunoblotting in ORF8-expressing Caco-2 cells with compound 1 (5 μM) treatment for 4 h. (**C**) The effect of compound 1 on IFN induction by Sendai virus (SeV) infection was determined by luciferase reporter assay using control (CTL) or CTPS1-depleted 293T cells treated with increasing concentrations of compound 1. (**D**) FLAG-CTPS1 expressed 293T cells were treated with compound 2 at the indicated concentrations for 2 h. CTPS1 was purified and subjected to binding analysis by in-gel fluorescence imaging and Coomassie blue staining. (**E and F**) Effect of compound 1 on intracellular CTP or CDP synthesis was determined by [amide-^15^N]glutamine tracing and mass spectrometry using SARS-CoV-2 ORF8-expressing Caco-2 cells (**E**) or SARS-CoV-2-infected Caco-2 cells (**F**) treated with increasing concentrations of compound 1. M + a indicates targeted metabolites labeled with [amide-^15^N]. Metabolites with M + 2 are below the detection limit. ND, not detected. (**G**) Effect of compound 1 on CTPS1 activity in CTP synthesis, in the presence of 2 mM ATP, 2 mM L-glutamine, 0.1 mM GTP, and increasing concentrations of UTP, was determined by *in vitro* enzymatic assay and analyzed by mass spectrometry. Inhibition of CTP synthesis was normalized to DMSO control. (**H through J**) Caco-2 cells were treated with compound 1 and infected with SARS-CoV-2 (MOI = 0.1). The mRNA abundance of antiviral genes was determined by real-time PCR at 48 h after SARS-CoV-2 infection (**H**). The effect of compound 1 on SARS-CoV-2 RNA abundance (**I**) and infectious viral progeny (**J**) was determined at 72 h after SARS-CoV-2 infection by real-time PCR analysis of total RNA and plaque assay of the medium, respectively. (**K**) The effect of compound 1 and its derivatives on SARS-CoV-2 replication was determined by plaque assay at 72 h post-infection (MOI = 0.1) in the medium of Caco-2 cells. (**L and M**) Caco-2 (**L**) and NHBE cells (**M**) were treated with the indicated compounds and infected with SARS-CoV-2 (MOI = 0.1). Viral titer in the medium was determined by plaque assay. Effects of these compounds on cell viability were determined by XTT assay and plotted. IC_50_, IC_90_, and CC_50_ were calculated. Data are presented as means ± SD of biological triplicates (**C, E, F, H through M**) and are representative of three independent experiments (B, D, and G). Statistical significance was calculated using the two-way ANOVA test or one-way ANOVA test. *P < 0.05; **P < 0.01; ***P < 0.001.

Inhibition of CTPS1 is expected to diminish CTP supply and restore IFN induction, thereby impeding SARS-CoV-2 replication. We thus sought to develop small-molecule inhibitors to further characterize the activity of CTPS1 in SARS-CoV-2 infection, which may enable an antiviral strategy. Because the GAT domain within CTPS1 has cysteine hydrolase activity and contains a catalytic cysteine (C399) in its active site, CTPS1 is sensitive to covalent inhibition by electrophilic compounds exemplified by 6-diazo-5-oxo-L-norleucine (DON) (26, 27). We thus screened a panel of electrophilic analogs to search for inhibitors of CTPS1. Our screening effort led to the identification of compound 1 as an effective CTPS1 inhibitor (Fig. 4A). As analyzed by two-dimensional gel electrophoresis, compound 1 demonstrated an inhibitory effect on IRF3 deamidation in Caco-2 cells expressing ORF8, and in A549 and LoVo cells expressing Nsp8 (Fig. 4B; Fig. S4A). Accordingly, compound 1 increased *IFNB1* expression in a dose-dependent manner in 293T cells infected with Sendai virus (Fig. S4B). To determine the specificity of compound 1, we depleted CTPS1 in 293T cells and examined IFN induction by a luciferase reporter assay. We found that compound 1 elevated IFN induction in control 293T cells, whereas it failed to do so in CTPS1-depleted 293T cells and had no effect on NF-κB activation, which is negatively regulated by CAD (Fig. 4C; Fig. S4C) (25). To validate that compound 1 targets CTPS1, we employed compound 2, a close relative of compound 1 containing a terminal alkyne tag, for biochemical labeling using 293T cells expressing CTPS1 (Fig. 4A). This assay showed that compound 2 covalently labeled CTPS1 in a dose-dependent manner, indicating direct binding between compound 1 and CTPS1 (Fig. 4D). Taken together, these results demonstrate that compound 1 inhibits CTPS1 to elevate IFN induction.

Activated CTPS1 also increases CTP supply in cells infected with SARS-CoV-2 to facilitate viral replication. With Caco-2 cells that stably express ORF8, we performed [amide-^15^N]glutamine flux analysis with compound 1 treatment. As shown in Fig. 4E, compound 1 treatment diminished [amide-^15^N]CTP and, to a much greater extent, [amide-^15^N]CDP in a dose-dependent manner. Similar reductions were observed in ORF8-expressing LoVo cells after compound 1 treatment (Fig. S4D). Remarkably, compound 1 potently diminished the intracellular concentration of [amide-^15^N]CTP and [amide-^15^N]CDP in Caco-2 cells infected with SARS-CoV-2 (Fig. 4F; Fig. S4E). These results show that an inhibitor of CTPS1 can block CTP synthesis in cells infected with SARS-CoV-2 or expressing ORF8. We then performed an *in vitro* biochemical assay to assess the effect of compound 1 on CTPS1 enzymatic activity. Compared with the DMSO group, compound 1 inhibited CTP synthesis by 55%–83% in the presence of low UTP concentration (Fig. 4G), whereas this inhibitory effect was diminished by increasing UTP concentration. This result indicates that compound 1 competes with UTP to bind CTPS1 to inhibit CTP synthesis.

To probe the biological consequence of compound 1, we analyzed the expression of antiviral genes, including *IFNB1*, *ISG56*, *CCL5*, and *Mx1* in SARS-CoV-2-infected Caco-2 cells. Real-time PCR analysis indicated that compound 1 at the concentrations of 2 µM and 6 µM elevated the expression of these antiviral genes (Fig. 4H). Consistent with the elevated antiviral gene expression, the abundance of viral RNAs, including *Nsp1*, *N*, and *E*, was reduced by compound 1 in a dose-dependent manner (Fig. 4I), with reduction of >50% and ~75% at the concentrations of 2 µM and 6 µM, respectively. The reduced viral RNA abundance also correlated with lower viral yield, in which compound 1 reduced viral yield by ~10-fold and 50-fold at the concentrations of 2 µM and 6 µM, respectively (Fig. 4J). Similar results were observed in SARS-CoV-2-infected NHBE cells when treated with compound 1, including elevated antiviral gene expression and reduced viral RNA and yield (Fig. S4F through H). Compound 1 treatment also significantly restored the inhibited expression of *IFNB1* and *ISG56* by Nsp8 and ORF8 (Fig. S4I). These results collectively show that compound 1 inhibits CTPS1 to impede nucleotide synthesis and restore IFN induction, which culminate in diminishing SARS-CoV-2 replication.

To improve the antiviral potency of compound 1, we designed and synthesized eight structural analogs, compounds 3–10. NMR and mass spectra of compounds 3–10 are consistent with their chemical structures. An IFN induction reporter assay showed that compounds 6 and 10 showed a better effect on IFN induction than compound 1 (Fig. S4J and K). Thus, we selected compounds 6 and 10 for further SARS-CoV-2 studies. Consistent with their ability to enhance IFN induction, compounds 6 and 10 showed more robust antiviral activity in SARS-CoV-2 infection than compound 1, as determined by plaque assay (Fig. 4K). These results identified a number of CTPS1 inhibitors that potently antagonize SARS-CoV-2 replication in cultured cells.

To evaluate the antiviral activity and cytotoxicity of CTPS1 inhibitors, we treated Caco-2, NHBE, Vero E6-hACE2, and Calu-3 cells with compounds 1, 6, and 10 and infected with SARS-CoV-2. Cytotoxicity of these compounds was determined using mock-infected cells. Our data showed that all three compounds displayed dose-dependent inhibition of viral replication (Fig. 4L and M; Fig. S4L and M). In Caco-2 cells, compound 1 inhibited SARS-CoV-2 replication with an IC50 of 0.17 µM and cell proliferation with a CC50 of 2.42 µM; compound 6 did so with an IC50 of 0.14 µM and CC50 of 2.82 µM; compound 10 more potently inhibited SARS-CoV-2 replication, with an IC50 of 0.11 µM and a CC50 of 6.17 µM (Fig. 4L). Similar results were also observed in NHBE cells (Fig. 4M), whereas these compounds exerted a similar effect on SARS-CoV-2 replication in Vero E6-hACE2 cells (Fig. S4L). By contrast, compounds 1 and 6 had IC50 values as high as their CC50 values, whereas compound 10 had a CC50 value that was 10-fold of its IC50 value in Calu-3 cells (Fig. S4M). These results show that all three compounds inhibited SARS-CoV-2 replication at concentrations much lower than their cytotoxic concentrations, and compound 10 displays the best antiviral activity and the least cytotoxicity.

### A CTPS1 inhibitor protects mice from SARS-CoV-2 infection

**Fig 5:**
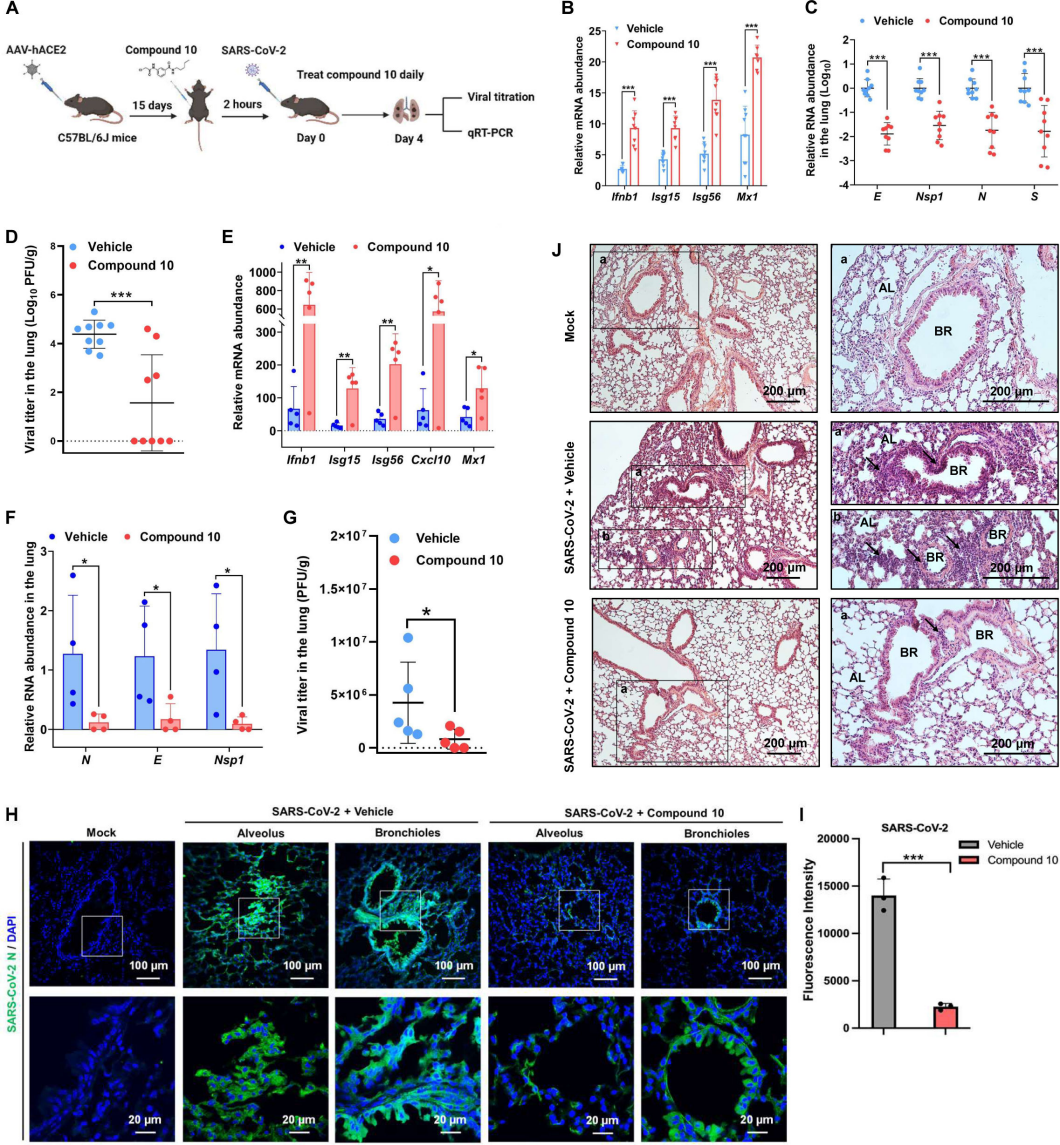
A CTPS1 inhibitor protects mice from SARS-CoV-2 infection. (**A**) Schematic of SARS-CoV-2 infection study in AAV-hACE2-transduced mouse model. (**B through D**) AAV-hACE2-transduced C57BL/6 J mice were intranasally infected with 1.5 × 10^5^ PFU of SARS-CoV-2 and treated with 35 mg/kg of compound 10 (*n* = 9) or vehicle (*n* = 9) daily by intraperitoneal injection. Mice were euthanized on 4 dpi, and lung tissues were harvested. Cellular antiviral gene expression (**B**) and RNA abundance of SARS-CoV-2 (**C**) were analyzed by real-time PCR. Viral titer in the lung homogenates was determined by plaque assay in Vero E6-hACE2 cells (**D**). (**E through J**) K18-hACE2 transgenic mice (*n* = 5 for each group) were pre-treated with compound 10 or vehicle control for 1 day and then infected with 1 × 10^4^ PFU of SARS-CoV-2 for 3 days. Compound treatment was performed daily. Innate immune gene expression (**E**) and viral RNA abundance (**F**) in the lung were analyzed by real-time PCR. Viral titer in the lung was determined by plaque assay in Vero E6-hACE2 cells (**G**). Immunofluorescence staining was performed on the frozen lung tissues with an antibody against SARS-CoV-2 N protein (**H and I**). Lung tissues were harvested, processed, and sectioned for H&E staining. AL, alveolus; BR, bronchioles; black arrows indicate inflammatory infiltrates (**J**). Data are presented as mean ± SD. Statistical significance was calculated using an unpaired, two-tailed Student’s *t*-test. *P < 0.05; **P < 0.01; ***P < 0.001.

Given the best antiviral activity and least cytotoxicity of compound 10 *in vitro*, we sought to determine the *in vivo* efficacy of compound 10 in two established mouse models of SARS-CoV-2 infection. Adeno-associated virus (AAV)-mediated expression of hACE2 in laboratory mice supports SARS-CoV-2 infection (28). We thus delivered AAV-hACE2 into C57BL/6 J mice and validated hACE2 expression via immunofluorescence staining (Fig. S5A). At 15 days post-AAV-hACE2 transduction, mice were infected with 1.5 × 10^5^ PFU of SARS-CoV-2 (Fig. 5A). The increasing viral titer and *Ifnb* mRNA levels in the lung indicated that the transduced mice support SARS-CoV-2 infection (Fig. S5B and C). Interestingly, there was no body weight loss during SARS-CoV-2 infection in the AAV-hACE2 mice model (Fig. S5D). Compound 10 treatment elevated the expression of cellular antiviral genes, including *Ifnb1*, *Isg15*, *Isg56,* and *Mx1*, at 4 days post-infection (dpi) (Fig. 5B). Conversely, compound 10 reduced the abundance of viral RNAs, including *E*, *Nsp1*, *N,* and *S*, by ~1.5 to 2 orders of magnitude in the lung (Fig. 5C). The reduced viral RNA abundance correlated with the decreased viral titer (Fig. 5D). K18-hACE2 transgenic mice are highly susceptible to SARS-CoV-2 infection and manifest with weight loss, lung inflammation, and mortality (29). To further assess the effect of compound 10 on SARS-CoV-2 replication, we infected K18-hACE2 transgenic mice with SARS-CoV-2, with or without compound 10 treatment. At 3 dpi, compound 10 elevated immune gene expressions and reduced the viral RNA abundance and viral titer in the lung compared with vehicle (Fig. 5E through G). Immunofluorescence staining further showed that robust viral N protein was detected in alveoli and bronchioli areas in the vehicle group at 3 dpi, although there was no body weight loss (Fig. 5H and I; Fig. S5E), whereas lower expression of viral protein was detected in the compound 10-treated group. To determine the effect of compound 10 on pathological features induced by SARS-CoV-2, we performed H&E staining with lung tissues. SARS-CoV-2 infection induced alveolar septal thickening and severe infiltration of inflammatory cells in alveoli and bronchioles that were widely distributed throughout the lung of the vehicle-treated mice (Fig. 5J; Fig. S5F). In compound 10-treated mice, weak and isolated infiltration of immune cells was detected along the bronchioles, and no marker of inflammation was detected in the alveolus. Collectively, these results indicate that compound 10 reduces SARS-CoV-2 replication and lung inflammation *in vivo*.

## DISCUSSION

The unprecedented COVID pandemic is caused by SARS-CoV-2, which rapidly evolved under the pressure of vaccine-induced neutralizing antibodies (30, 31). Studies involving cultured cells, model animals, and COVID-19 patients indicate that SARS-CoV-2 effectively inhibits the production of type I and III interferons (10, 32, 33). However, the molecular mechanism by which SARS-CoV-2 does so is not well understood. Here, we report that SARS-CoV-2 deploys multiple proteins to activate CTPS1, which promotes CTP synthesis while inactivating IRF3 to mute IFN induction. Remarkably, pharmacological inhibition of CTPS1 potently impedes CTP synthesis and effectively restores IFN induction, thereby diminishing SARS-CoV-2 replication. This study offers the proof of principle to develop an antiviral strategy targeting a host enzyme.

The dysregulated immune response is a characteristic shared among COVID-19 patients under severe and critical conditions (34, 35). Among the skewed cytokine profile, type I IFNs and inflammatory cytokines are produced at very low or under detection levels in most severe or critical COVID-19 patients, which likely contributes to the rapid replication of SARS-CoV-2 in these patients (36). To dissect the mechanism of innate immune evasion by SARS-CoV-2, we first showed that RNA produced from SARS-CoV-2-infected NHBE cells potently induced IFN, whereas SARS-CoV-2 infection failed to do so compared with Sendai virus infection, suggesting that SARS-CoV-2 viral proteins antagonize IFN induction. Indeed, a screen utilizing a SARS-CoV-2 expression library identified ORF7b, ORF8, Nsp8, and Nsp13 as inhibitors of IFN induction. Further analysis showed that these SARS-CoV-2 polypeptides target IRF3 for post-translational modification. Given the ubiquitous role of type I IFNs in the host innate immune defense against viral infection, regulatory mechanisms governing IRF3 activation are expected to operate independently of cell type and tissue origin (37, 38). Upstream components, such as pattern recognition receptors and their cognate adaptors, may be tissue- and cell type-specific (39, 40). Accordingly, viral factors targeting these upstream components likely function in a tissue-dependent manner, in contrast to those meddling with the downstream components in all cell types. In the lung, the epithelial cells and pneumocytes of the airway and respiratory tract are the first responders in IFN production during SARS-CoV-2 infection (32, 41).

Nucleotide supply is a rate-limiting factor for cell proliferation and virus replication (42, 43). As intracellular obligate pathogens, viruses rely on cellular machinery for their macromolecular biosynthesis (14, 44). During viral productive infection, nucleotides are used for transcription, translation (ribosome regeneration), genome replication, and lipid synthesis for the assembly and maturation of virion progeny. Not surprisingly, viruses often activate metabolic enzymes to fuel nucleotide synthesis in support of their replication (45). We discovered that SARS-CoV-2 infection and specifically ORF7b and ORF8 activate CTPS1 to promote *de novo* CTP synthesis, thereby fueling viral replication. Surprisingly, activated CTPS1 also inhibits type I IFN induction via deamidating IRF3. Deamidation results in the loss of DNA-binding activity of IRF3 to its cognate sequences, thereby diminishing IFN induction. Thus, SARS-CoV-2 couples the inhibition of type I IFN induction to CTP synthesis via activating CTPS1. This predicts that SARS-CoV-2 relies on CTPS1 for its replication, and conversely, inhibiting CTPS1 likely impedes SARS-CoV-2 replication. Indeed, we found that depletion and pharmacological inhibition of CTPS1 greatly diminished CTP synthesis and effectively restored antiviral IFN induction in SARS-CoV-2 infection.

The newly developed small-molecule inhibitors exhibited good specificity in targeting CTPS1. They stimulate IFN induction, but not NF-ĸB activation. CTPS1 and CAD negatively regulate IRF3 and NF-ĸB activation, respectively (21, 46). Furthermore, the effect of compound **1** on IFN induction was observed in wild-type cells but not in CTPS1-depleted cells, supporting the conclusion that compound 1 inhibits CTPS1 to boost IFN production. Indeed, a derivative of compound 1 carries a reactive warhead that cross-links with CTPS1 *in vitro*. Compound 10, a derivative of compound 1, potently reduced nucleotide levels and restored IFN induction in two mouse models used for SARS-CoV-2 infection, thereby impeding SARS-CoV-2 replication and pathogenesis. Thus, these compounds target CTPS1 to promote IFN induction while depleting nucleotide supply, which is distinct from other nucleotide-nucleoside analogs (47). However, we cannot exclude the possibility that these small molecules target cellular proteins other than CTPS1. Nevertheless, considering that CTPS1 is essential for cell proliferation, CTPS1 inhibitors potentially induce toxicity in proliferating cells. The premise is that SARS-CoV-2 activates CTPS1 to facilitate its replication, which permits the selective inhibition of CTPS1 with low concentrations of these small-molecule inhibitors. Additionally, CTPS1 negatively regulates innate immunity in a CTP-independent manner, although it is required for CTP synthesis during lymphocyte proliferation (21, 48). Thus, supplementing CTP to CTPS1-inhibited mice is expected to improve the antiviral immune effect of the CTPS1-targeted therapy. Such a strategy would enhance interferon production without compromising immune cell proliferation. Conceivably, the application of CTPS1 inhibitors to COVID patients has to be optimized to avoid toxicity to bystander cells that are vital for host immune defense, such as T cells. These findings provide a proof of concept to target CTPS1 for antiviral therapy against SARS-CoV-2.

## MATERIALS AND METHODS

### Cell culture

HEK293T, A549, LoVo, *Irf3^−/−^Irf7^−/−^* mouse embryonic fibroblasts (MEFs) were cultured in Dulbecco’s modified Eagle’s medium (DMEM; Hyclone). Caco-2 and Calu-3 cells were cultured in a minimal essential medium (MEM). All cell lines were supplemented with 10% fetal bovine serum (FBS; HyClone), penicillin (100 U/mL), and streptomycin (100 µg/mL) and maintained at 37°C in a humidified atmosphere of 5% CO_2_. Primary normal, human bronchial/tracheal epithelial (NHBE) cells were cultured in an airway epithelial cell medium according to ATCC’s recommendation.

### Mice

C57BL/6 J mice (8–10 weeks of age) and hemizygous K18-hACE2 mice (8–10 weeks of age) [strain B6.Cg-Tg(K18-ACE2)2Prlmn/J, 034860] were purchased from the Jackson Laboratory. Mice were housed in individually ventilated racks equipped with HEPA filters, supplied with regular diet and sterilized water.

### Viruses

Sendai virus (SeV) was purchased from Charles River. SARS-CoV-2 was propagated in Vero E6-hACE2 cells. All SARS-CoV-2-related propagation, infection, and titration were performed in the biosafety level 3 (BSL-3) facility (USC).

#### SARS-CoV-2 propagation

Vero E6-hACE2 cells were seeded at 1.5 × 10^6^ cells per T25 flask for 12 h. Cells were washed with FBS-free DMEM once and infected with SARS-CoV-2 at MOI of 0.005 in FBS-free DMEM. Cells were monitored daily for cytopathic effect (CPE). When virus-induced CPE reached approximately 80% (around 72 h after viral infection), the virus-containing medium was harvested, centrifuged at 3,000 rpm for 5 min, and stored at −80°C.

#### SARS-CoV-2 infection

NHBE (1.5 × 10^5^ cells), Calu-3 (5 × 10^5^ cells), or Caco-2 cells (2 × 10^5^ cells) were seeded in one well of 12-well plates. Cells were washed with FBS-free medium before viral infection. SARS-CoV-2 was diluted in 250 µL (per well) medium and added to the above cells. Viral infection was incubated on a rocker for 45 min at 37°C. Cells were washed with fresh medium, and medium containing 10% FBS was added.

#### SARS-CoV-2 viral titration (plaque assay)

Vero E6-hACE2 cells were seeded in 6- or 12-well plates. When cell confluence reached 100%, cells were washed with FBS-free medium and infected with medium containing serially diluted SARS-CoV-2. After infection, the medium was removed, and overlay medium containing FBS-free 1× DMEM and 1% low-melting point agarose was added. At 72 h post-infection, cells were fixed with 4% paraformaldehyde (PFA) overnight and stained with 0.2% crystal violet. Plaques were counted on a lightbox.

### Plasmids

Luciferase reporter plasmids for IFN-β, NF-κB promoters, RIG-I-N, MAVS, TBK1, IRF3-5D, and shRNA for human CTPS1 were described previously (46, 49, 50). pLVX-EF1alpha-2XStrep-IRES-Puro containing SARS-CoV-2 viral genes described previously and provided by Dr. Nevan J. Krogan (22).

### Quantitative real-time PCR (qRT-PCR)

qRT-PCR was performed as previously described (46). Briefly, total RNA was extracted from mock- or virus-infected cells using TRIzol reagent (Invitrogen). cDNA was synthesized from total RNA (1 µg) using reverse transcriptase (Invitrogen) according to the manufacturer’s instruction. Quantitative real-time PCR (qRT-PCR) reaction was performed with SYBR Green Master Mix (Sigma) or qPCRBIO SyGreen Blue Mix Lo-ROX (Genesee Scientific). Gene expression level was calculated by the 2^−ΔΔCt^ method. Primers for qRT-PCR were listed in Table S1.

### Lentivirus-mediated stable cell line construction

Lentivirus production was carried out in HEK293T cells. Briefly, 293T cells were co-transfected with packaging plasmids (VSV-G, DR8.9) and pCDH lentiviral expression vector or lentiviral shRNA plasmids. At 48 h post-transfection, the medium was harvested and filtered. HEK293T, *Irf3^−/−^Irf7^−/−^* MEFs, Caco-2, LoVo, and A549 cells were infected with the virus-containing medium, supplied with polybrene (8 µg/mL), and centrifuged at 1,800 rpm for 50 min at 30°C. Cells were incubated at 37°C for 6 h and maintained in DMEM with 10% FBS. Selection was performed at 48 h post-infection with puromycin (1–2 μg/mL) or hygromycin (200 µg/mL).

### Dual-luciferase reporter assay

HEK293T cells in 24-well plates (~50% cell density) were transfected with a reporter plasmid cocktail containing 50 ng luciferase reporter plasmid (ISRE-luc, IFN-β-luc, or NF-κB), 5 ng TK-renilla luciferase reporter (control vector), and the indicated expression plasmids by calcium phosphate precipitation. Whole cell lysates (WCLs) were prepared at 24–30 h post-transfection and used for dual luciferase assay according to the manufacturer’s instruction (Promega).

### CRISPR-Cas9-mediated genome editing

To establish CTPS1-knockout cell lines, single-guide RNAs (sgRNAs) targeting CTPS1 were designed. The annealed sgRNA oligonucleotides were inserted into LentiCRISPRv2-Hygro vector. Lentivirus was produced as described above. Caco-2 cells were transduced with lentivirus expressing sgRNAs targeting CTPS1 and selected with 1 µg/mL hygromycin. Single colonies were isolated and screened by immunoblotting with CTPS1 antibody. sgRNA sequence targeting CTPS1 was listed in Table S1.

### Protein expression and purification

HEK293T cells were transfected with FLAG-tagged or GST-tagged genes of interest. Cells were harvested at 48 h post-transfection and lysed with Triton X-100 buffer (20 mM Tris, pH 7.5, 150 mM NaCl, 1 mM EDTA, 20 mM β-glycerophosphate, 10% glycerol) supplemented with a protease inhibitor cocktail (Roche). WCLs were sonicated, incubated at 4°C for 30 min on a rotator, and centrifuged at 12,000 rpm for 30 min. Supernatant was filtered and precleared with Sepharose 4B agarose beads (Thermo) at 4°C for 1 h. The pre-cleared lysates were incubated with anti-FLAG M2 agarose beads or glutathione-conjugated agarose beads at 4°C for 4 h. Anti-FLAG M2 magnetic beads were washed extensively with lysis buffer and eluted with 0.2 mg/mL 3× FLAG peptide. Glutathione-conjugated agarose beads were extensively washed and used immediately for *in vitro* on-column deamidation assay. Concentration of purified proteins was analyzed by SDS-PAGE and Coomassie blue staining, with BSA as a standard.

### Two-dimensional gel electrophoresis

Cells (1 × 10^6^) were resuspended in 150 µL rehydration buffer (8 M urea, 2% CHAPS, 0.5% IPG buffer, 0.002% bromophenol blue), sonicated three times, and incubated on ice for 15 min. WCLs were centrifuged at 12,000 × *g* for 15 min. The cleared lysate was loaded to IEF strips for isoelectric focusing with a program comprising: 20 V, 10 h (rehydration); 500 V, 1 h; 1,000 V, 1 h; 1,000–5,000 V, 4 h; 5,000 V, 4 h. Then, strips were incubated with SDS equilibration buffer (50 mM Tris-HCl [pH 8.8], 6 M urea, 30% glycerol, 2% SDS, and 0.001% bromophenol blue) containing 10 mg/mL DTT for 15 min and SDS equilibration buffer containing 2-iodoacetamide for 15 min. Strips were washed with SDS-PAGE buffer, resolved by SDS-PAGE, and analyzed by immunoblotting.

### *In vitro* deamidation assay

To analyze the effects of SARS-CoV-2 ORF7b, ORF8, and Nsp8 on CTPS1-mediated IRF3 deamidation, plasmids containing ORF8 and Nsp8 were transfected into 293T/FLAG-CTPS1 cells. Cell lysate was prepared at 48 h post-transfection, and proteins were purified with anti-FLAG M2 agarose (Sigma). *In vitro* on-column deamidation of IRF3 was performed as previously reported (46). Briefly, 0.2 µg of CTPS1 and 0.6 µg of IRF3-GST (on beads) were added to a total volume of 50 µL. The reaction was carried out at 37°C for 45 min in deamidation buffer (50 mM Tris-HCl at pH 8.0, 20 mM MgCl_2_, 5 mM KCl, 1 mM ATP, 1 mM GTP). IRF3-GST was eluted with rehydration buffer (8 M Urea, 2% CHAPS, 0.5% IPG buffer, 0.002% bromophenol blue) at room temperature. Samples were analyzed by two-dimensional gel electrophoresis and immunoblotting.

### Metabolite profiling and isotope tracing

Caco-2 cells were mock-infected or infected with SARS-CoV-2 at MOI = 1. Cells were harvested at 6, 24, 48, and 72 h post-infection for metabolite analysis. Isotope-tracing experiments were performed as previously described (46). To analyze the effect of SARS-CoV-2 proteins on nucleotide synthesis, LoVo and Caco-2 cells stably expressing SARS-CoV-2 ORF7b, ORF8, and Nsp8 were cultured with medium containing [amide-^15^N]glutamine for 30 min and 60 min. Cells were washed with 1 mL ice-cold ammonium acetate (NH_4_AcO, 150 mM, pH 7.3), added 1 mL −80°C cold MeOH, and incubated at −80°C for 20 min. After incubation, cells were scraped off and transferred into microfuge tubes. Samples were pelleted at 4°C for 5 min at 15,000 rpm. The supernatant was transferred into new microfuge tubes, dried at room temperature under vacuum, and re-suspended in water for LC-MS run.

Samples were randomized and analyzed on a Q-Exactive Plus hybrid quadrupole-Orbitrap mass spectrometer coupled to Vanquish UHPLC system (Thermo Fisher). The mass spectrometer was run in polarity switching mode (+3.00 kV/−2.25 kV) with an *m*/*z* window ranging from 65 to 975. Mobile phase A was 5 mM NH_4_AcO, pH 9.9, and mobile phase B was acetonitrile. Metabolites were separated on a Luna 3 µm NH_2_ 100 NH_2_ 100 Å (150 × 2.0 mm) column (Phenomenex). The flow rate was 0.3 mL/min, and the gradient was from 15% A to 95% A in 18 min, followed by an isocratic step for 9 min and re-equilibration for 7 min. All samples were run in biological triplicate. Metabolites were detected and quantified as area under the curve based on retention time and accurate mass (five ppm) using the TraceFinder 4.1 (Thermo Scientific) software. Raw data were corrected for naturally occurring ^15^N abundance.

### CTPS1 enzymatic activity assay

FLAG-CTPS1-expressing 293T stable cells were transfected with plasmids containing SARS-CoV-2 ORF7b, ORF8, and empty vector for 48 h. CTPS1 was purified with anti-FLAG M2 agarose via one-step affinity chromatography for *in vitro* assay. CTPS1 activity was determined by measuring the conversion of UTP to CTP via mass spectrometry. The standard reaction mixture containing 50 mM Tris-HCl (pH 8.0), 10 mM MgCl_2_, 10 mM 2-mercaptoethanol, 2 mM L-glutamine, 1 mM GTP, 1 mM ATP, increasing concentrations of UTP (0 to 30 mM), and an appropriate dilution of CTPS1 in a total volume of 50 µL. The reactions were equilibrated to 37°C for 45 min and quenched by adding 250 µL cold (−80°C) methanol and incubating at −80°C for 20 min. The metabolites were analyzed by LS-MS as described above.

To determine the effect of compound 1 on CTPS1 enzymatic activity, an *in vitro* enzymatic activity assay was performed with purified CTPS1 and diluted compound 1. In brief, 10 µM compound 1 or DMSO (control) was added to the reaction mixture, which contained 50 mM Tris-HCl (pH 8.0), 10 mM MgCl_2_, 10 mM 2-mercaptoethanol, 2 mM L-glutamine, 0.1 mM GTP, 1 mM ATP, increasing concentrations of UTP (0 to 16 mM), and an appropriate dilution of CTPS1. The reaction was carried out at 37°C for 45 min. The samples were analyzed by LC-MS.

### Small molecule synthesis

Reagents and solvents were obtained from commercial suppliers and used without further purification unless otherwise stated. Flash column chromatography was carried out using an automated system (Teledyne Isco CombiFlash). Reverse-phase high-performance liquid chromatography (RP-HPLC) was carried out on a Shimadzu HPLC system. All anhydrous reactions were carried out under a nitrogen atmosphere. NMR spectra were obtained on Varian VNMRS-500, VNMRS-600, or Mercury-400.

#### Step 1

X (1 eq.) and Y (1.2 eq.) were dissolved in dichloromethane (DCM)/dimethylformamide (DMF) (4:1) (Table 1). Hexafluorophosphate benzotriazole tetramethyl uronium (HBTU) (4 eq.) and triethylamine (4 eq.) were added, and the solution was allowed to stir at room temperature for 16 h. After 16 h, the reaction mixture was diluted with EtOAc and washed with 10% Na_2_CO_3_ followed by brine three times. The organic layer was dried with Na_2_SO_4_ and concentrated by rotary evaporation. The residue was purified via flash column chromatography (EtOAc/Hex). All products moved to step 2.

**TABLE 1:** Synthesis of target compounds

Compound	X	Y
1	3-Nitrobenzoic acid	m-Anisidine
2	3-Nitrobenzoic acid	3-(Prop-2-ynyloxy)aniline
6	3-Nitrobenzoic acid	o-Anisidine
10	3-Nitrobenzoic acid	Amylamine

#### Step 2

Intermediates from step 1 were dissolved in MeOH. Zn (5 eq.) and NH_4_Cl (5 eq.) were added, and the mixture was allowed to stir at room temperature for 16 h. The reaction mixture was dissolved in EtOA and washed with 10% Na_2_CO_3_ followed by brine three times. The organic layer was dried over NaSO_4_ and concentrated by rotary evaporation to yield the intermediates that were used in the next reaction without further purification.

#### Step 3

Intermediates from step 2 (1 eq.) were dissolved in anhydrous DCM/THF (1:4) under nitrogen gas. DIPEA (1.2 eq) was added via a syringe. Chloroacetyl chloride (1.2 eq) was added via syringe slowly dropwise. The reaction mixture was allowed to be stirred overnight. The mixture was diluted with EtOAc and washed with 10% Na_2_CO_3_. The organic layer was dried over NaSO_4_, and the residue was purified via flash chromatography (EtOAc/hexane) to yield the final products.

### Compound treatments

For SARS-CoV-2 infection, Caco-2 or NHBE cells were pre-treated with compound 1 or its derivatives for 2 h, after which the medium was removed. Cells were washed and infected with SARS-CoV-2. Then, the medium containing the virus was removed, and the cells were cultured with the compound-containing medium. Compounds were added daily during viral infection to the end of the experiments, with DMSO used as a control. To test the effect of compound 1 on IRF3 deamidation, Caco-2 cells expressing ORF8, or A549 and LoVo cells expressing Nsp8 were treated with 5 µM compound 1 for 4 h. To analyze the effect of compound 1 on intracellular metabolites, ORF8-expressing Caco-2 or LoVo cells were treated with 5 µM compound 1 for 2 h, and cells were prepared for metabolite extraction.

### Determine the inhibitory and cytotoxicity concentrations of compounds

Caco-2, NHBE, Calu-3, and Vero E6-hACE2 cells were pre-treated with compound 1, 6, or 10 and infected with SARS-CoV-2 (MOI = 0.1 for Caco-2, NHBE, and Calu-3; MOI = 0.001 for Vero E6-hACE2). Compounds were added daily during viral infection until the end of the experiments (72 h for Caco-2 and Calu-3, 48 h for NHBE, and 24 h for Vero E6-hACE2), with DMSO used as a control. Viral titer in the medium was determined by plaque assay. Cytotoxicity assay was performed for uninfected Caco-2, NHBE, Calu-3, and Vero E6-hACE2 cells with the same treatment schedule as that of viral replication assay. Cytotoxicity was determined with an XTT assay kit (Roche), according to the manufacturer’s instructions. The percentage of inhibition was normalized to DMSO control. IC50, IC90, and CC50 were calculated with GraphPad Prism software.

### Antiviral study of compound 10 in mouse models

Adeno-associated virus (AAV)-mediated human ACE2 (hACE2) expressing mice and K18-hACE2 transgenic mice were selected to evaluate the *in vivo* antiviral efficacy of compound 10 in SARS-CoV-2 infection.

C57BL/6 J mice were intranasally infected with AAV-CMV-hACE2 virus (5 × 10^10^ genomic copies per mouse) for 15 days. Mice were intraperitoneally administered with compound 10 that was diluted in 100 µL vehicle containing 55% PEG, 30% PBS, and 15% Tween-80 at a dose of 35 mg per kg body weight, 2 h before intranasal infection with 1.5 × 10^5^ PFU of SARS-CoV-2 in 30 µL PBS. Compound 10 was administered daily. At 4 days post-infection (dpi), mice were humanely euthanized. Whole right lungs were harvested and homogenized in FBS-free DMEM for viral titration via plaque assay, and the left lungs were used for RNA extraction and real-time PCR analysis.

K18-hACE2 transgenic mice were intraperitoneally administered with compound 10 at a dose of 50 mg/kg and vehicle 1 day before infection with 1 × 10^4^ PFU of SARS-CoV-2. Body weight and compound administration were monitored daily. Mice were humanely euthanized at 3 dpi, and the lung tissues were harvested for viral titration, real-time PCR, and pathological analysis.

All mice were anesthetized with 3.5% isoflurane for viral infection and compound administration. All antiviral studies were performed in an animal biosafety level 3 (ABSL3) facility at USC. All experiments were conducted under protocols approved by the IACUC.

### Histology and immunofluorescence analysis

K18-ACE2 transgenic mice were euthanized on day 3 after SARS-CoV-2 infection. Fresh lung pieces were fixed with 10% formalin for 24 h. The post-caval lobes of the left lung were sent to the histology lab in USC of the School of Pharmacy Core Facilities for processing and hematoxylin and eosin (H&E) staining. In brief, tissues were dehydrated and embedded in paraffin. Paraffin-embedded tissue blocks were sectioned with 5 µm thickness. Sections were processed by H&E staining and imaged by ZEISS Axio Scope.A1 microscope (Zeiss) and analyzed by ZEISS ZEN Blue software.

Immunofluorescence staining was performed on the middle lobes of the left lung fixed with 10% formalin. Tissues were embedded in optimal cutting temperature (OCT) compound and frozen immediately at −80°C. Subsequently, the frozen tissues were cut into 8 µm sections using a cryostat-microtome. Sections were blocked with 10% normal goat serum diluted in PBST for 1 h and incubated with primary antibodies diluted in 10% normal goat serum overnight at 4°C. After washing with PBST, sections were incubated with species-matched secondary antibodies for 30 min at room temperature. Then tissue sections were washed with PBST, mounted with VectaMount Mounting Medium (Vector Laboratories), and analyzed by a confocal microscope (Nikon).

### Statistical analysis

Statistical analyses were performed using GraphPad Prism software to perform Student’s *t*-test or analysis of variance (ANOVA) on at least three independent replicates. *P* values of <0.05 were considered statistically significant for each test.

## SUPPLEMENTAL MATERIAL

Supplemental material mbio.00649-25-s0001.pdf Fig. S1-S5; Table S1.

ASM does not own the copyrights to Supplemental Material that may be linked to, or accessed through, an article. The authors have granted ASM a non-exclusive, world-wide license to publish the Supplemental Material files. Please contact the corresponding author directly for reuse.
