# Supplementary material for: Targeting CTP synthetase 1 to restore interferon induction and impede nucleotide synthesis in SARS-CoV-2 infection
Source: mBio. 2025 Apr 29;16(6):e00649-25. doi: 10.1128/mbio.00649-25 (PMC12153265; doi:10.1128/mbio.00649-25)
Supplement: Supplemental material — Fig. S1-S5; Table S1. [file mbio.00649-25-s0001.pdf]

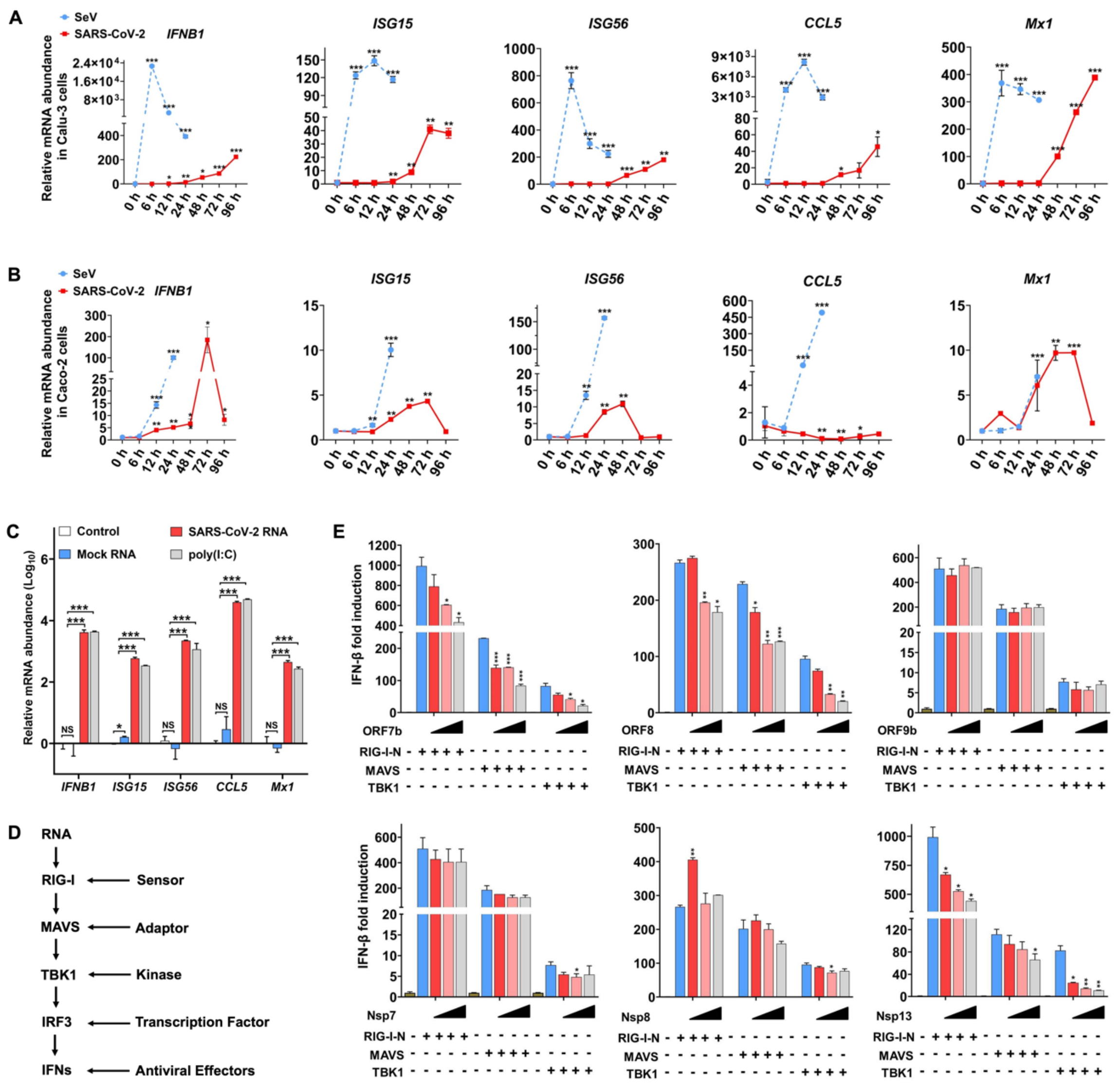

Fig S1 SARS-CoV-2 inhibits IFN induction. (A and B) Calu-3 and Caco-2 cells were infected with Sendai virus (SeV) (100 HAU/ml) or SARS-CoV-2 (MOI = 1). Total RNA was extracted, reverse transcribed and analyzed by real-time PCR with primers specific for *IFNB1*, *ISG15*, *ISG56*, *CCL5* and *Mx1*. (C) NHBE cells were transfected with poly(I:C) and RNA isolated from mock- or SARS-CoV-2-infected NHBE cells (MOI = 1, 72 hpi). RNA extraction and real-time PCR were performed as in (A and B). (D) Diagram of the RIG-I-IFN pathway that senses

double-stranded RNA. (E) Effect of SARS-CoV-2 proteins on IFN-β induction was determined by luciferase reporter assay in 293T cells transfected with an IFN-β reporter cocktail, increasing amounts of plasmids containing SARS-CoV-2 genes and components of the RIG-I-IFN pathway. Data are presented as means ± SD of biological triplicates (A, B, C, and E). Statistical significance was calculated using the one-way ANOVA test or unpaired, two-tailed Student's *t*-test. *$P < 0.05$; **$P < 0.01$; ***$P < 0.001$.

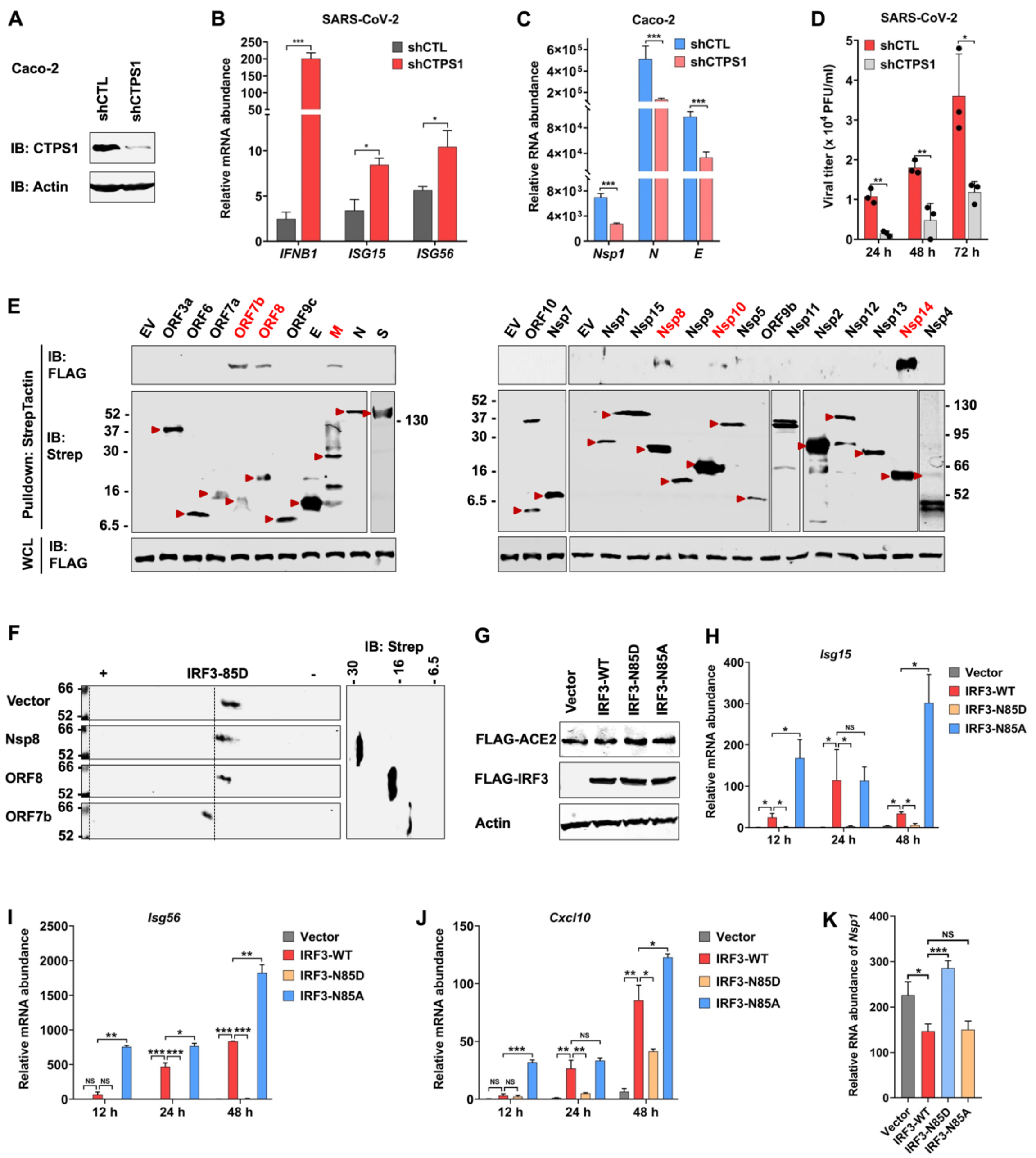

Fig S2 SARS-CoV-2 promotes CTPS1-mediated IRF3 deamidation to impede IFN induction. (A) Knockdown of CTPS1 was determined by immunoblotting using Caco-2 cells infected with lentivirus containing control (CTL) or CTPS1 shRNA. (B) Effect of CTPS1 depletion on the expression of cellular antiviral genes was determined by real-time PCR at 24 h post SARS-CoV-2 (MOI = 0.5) infection in

control and CTPS1-depleted Caco-2 cells. (C and D) Control and CTPS1-depleted Caco-2 cells were infected with SARS-CoV-2 (MOI 0.1) for 72 h. Effect of CTPS1 depletion on viral gene expression was determined by real-time PCR analysis using total RNA (C). Viral titer in the medium was measured by plaque assay in Vero E6-hACE2 cells (D). (E) Interactions between CTPS1 and SARS-CoV-2 proteins were analyzed by co-immunoprecipitation in transfected 293T cells. Strep is a tag for SARS-CoV-2 proteins. (F) Effect of SARS-CoV-2 proteins on the charge status of IRF3-N85D was determined by two-dimensional gel electrophoresis and immunoblotting in IRF3-N85D knock-in 293T cells. (G) $Irf3^{-/-}/Irf7^{-/-}$ MEFs were infected with lentivirus containing FLAG-tagged human ACE2, selected with hygromycin, then reconstituted with Vector, IRF3-WT, IRF3-N85D and IRF3-N85A. Expression levels of hACE2 and IRF3 were analyzed by immunoblotting. (H-K) Effect of IRF3 and its mutants on indicated antiviral gene expression (H-J) and SARS-CoV-2 RNA abundance (K) was assessed by real-time PCR with total RNA extracted at 24 h after SARS-CoV-2 infection (MOI =0.01). Data are presented as means ± SD of biological triplicates (B-D, H-K) and are representative of three independent experiments (A, E-G). Statistical significance was calculated using the two-way ANOVA test, one-way ANOVA test, or unpaired two-tailed Student's *t*-test. *$P < 0.05$; **$P < 0.01$; ***$P < 0.001$.

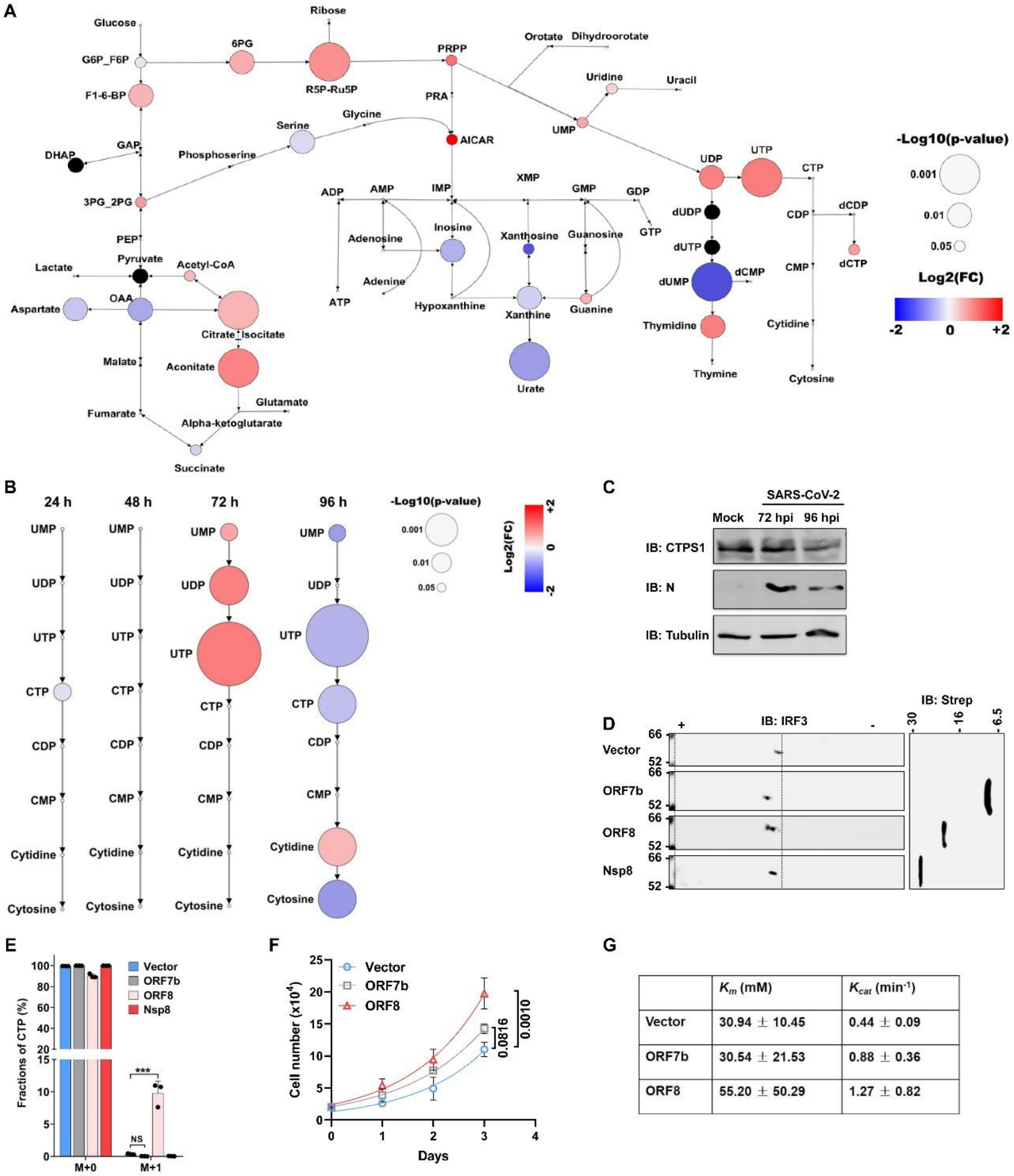

Fig S3 SARS-CoV-2 polypeptides increase CTPS1 activities. (A) Metabolites of the central carbon metabolism in Caco-2 cells altered by SARS-CoV-2 infection (MOI=0.1) were shown as metabolic map (normalized with mock infected group). Black circles represent undetectable metabolites. (B) Indicated pyrimidines in Caco-2 cells altered by SARS-CoV-2 infection (MOI=0.1) were

shown as metabolic map (normalized with mock infected group). (C) Caco-2 cells were infected with or without SARS-CoV-2 for 72 and 96 hours. CTPS1 and N protein levels were determined by immunoblotting. (D) Effect of SARS-CoV-2 proteins on IRF3 charge status was determined by two-dimensional gel electrophoresis and immunoblotting using lysates of Caco-2 cells stably expressing indicated viral proteins. (E) Effect of SARS-CoV-2 proteins on intracellular CTP synthesis was determined by [amide-^{15}N]glutamine tracing and mass spectrometry by using SARS-CoV-2 ORF7b, ORF8 and Nsp8 expressing LoVo cells. M+a indicates targeted metabolites labeled with [amide-^{15}N]. (F) Proliferation of Caco-2 cells stably expressing ORF7b or ORF8 was determined by cell number counting. (G) Effect of SARS-CoV-2 ORF7b and ORF8 on CTPS1 activity in CTP synthesis was determined by *in vitro* enzymatic assay. Data are presented as means ± SD of biological triplicates (A, B, E, and F) and are representative of three independent experiments (C, D, and G). Statistical significance was calculated using the one-way ANOVA test or unpaired, two-tailed Student's *t*-test. ***$P < 0.001$; NS, not significant.

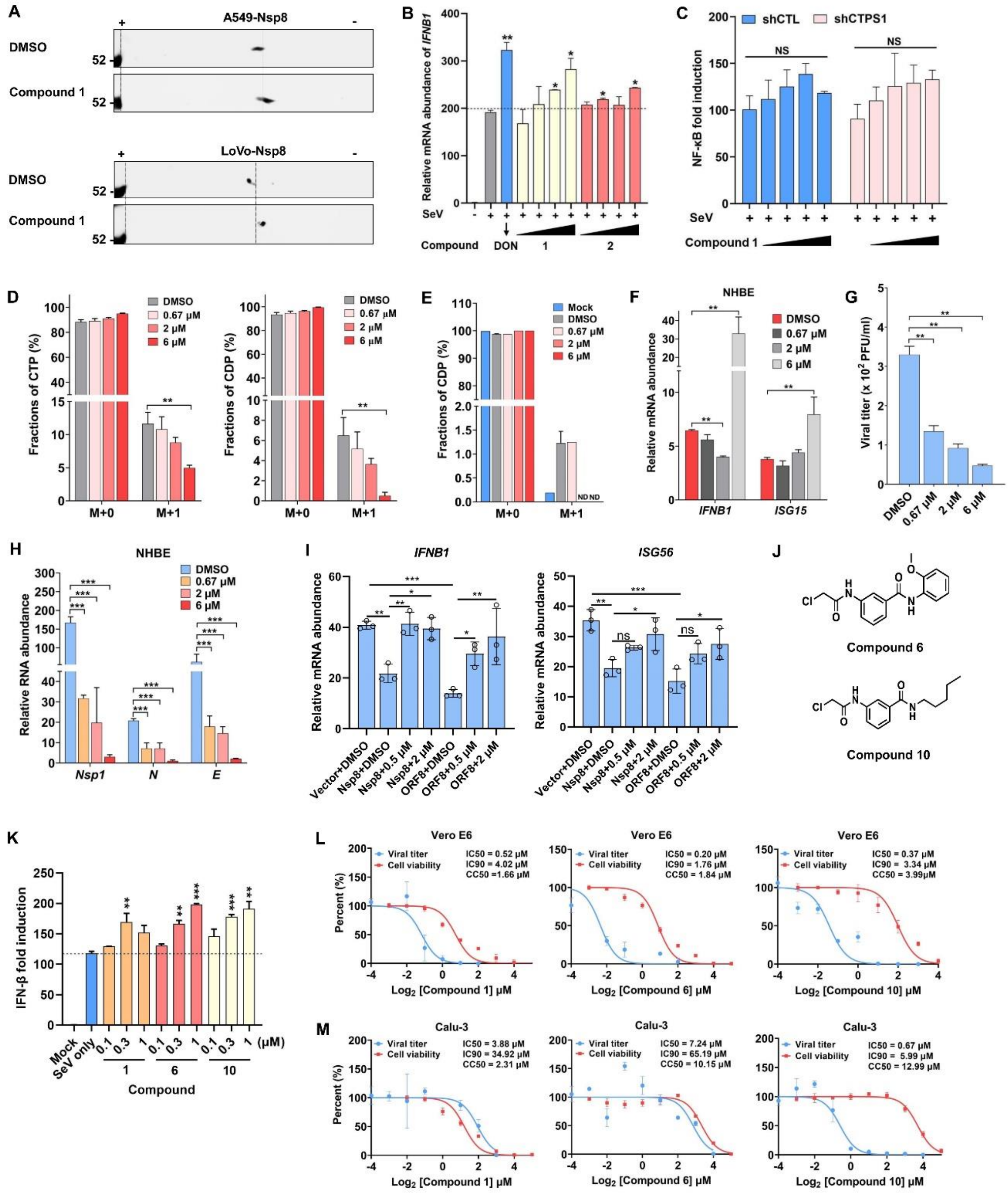

Fig S4 CTPS1 inhibitors impede SARS-CoV-2 replication. (A) Effect of compound **1** on SARS-CoV-2 Nsp8-induced IRF3 deamidation was analyzed by two-dimensional gel electrophoresis and immunoblotting in Nsp8-expressed A549 or LoVo cells with compound **1** (5 μM) treatment for 4 h. (B) Effect of compound **1**

on *IFNB1* expression in in 293T cells infected with Sendai virus was determine by real-time PCR analysis. (C) Effect of compound **1** on NF-KB activation by Sendai virus (SeV) infection was determined by luciferase reporter assay using control (CTL) or CTPS1-depleted 293T cells treated with increasing concentrations of compound **1**. (D) Effect of compound **1** on intracellular CTP or CDP synthesis was determined by [amide-^{15}N]glutamine tracing and mass spectrometry using SARS-CoV-2 ORF8-expressed LoVo cells treated with increasing concentrations of compound **1**. (E) Effect of compound **1** on intracellular CDP synthesis was determined by [amide-^{15}N]glutamine tracing and mass spectrometry using SARS-CoV-2-infected Caco-2 cells treated with increasing concentrations of compound **1**. ND, not detected. (F-H) NHBE cells were treated with compound **1** and infected with SARS-CoV-2. The mRNA abundance of antiviral genes was determined by real-time PCR (F). Effects of compound **1** on SARS-CoV-2 infectious viral progeny (G) and RNA abundance (H) were determined by plaque assay and real-time PCR, respectively. (I) 293T cells were transfected with empty vector, Nsp8, or ORF8 and treated with Compound **1** followed by infection with SeV. The mRNA abundance of antiviral genes was determined by real-time PCR at 6 h after infection. (J) Structures of compound **6** and **10**. (K) Effect of compound **1**, **6**, and **10** on IFN-β induction by Sendai virus (SeV) infection was determined by luciferase reporter assay using 293T cells treated with increasing concentrations of compounds. (L and M) Vero E6 (L) and Calu-3 cells (M) were treated with the indicated compounds and infected with SARS-

CoV-2 (MOI = 0.1). Viral titer in the medium was determined by plaque assay. Effects of these compounds on cell viability were determined by XTT assay and plotted. IC_{50}, IC_{90} and CC_{50} were calculated. Data are presented as means ± SD of biological triplicates (B-I, K-M) and are representative of three independent experiments (A). Statistical significance was calculated using the two-way ANOVA test or one-way ANOVA test. *$P < 0.05$; **$P < 0.01$; ***$P < 0.001$.

.

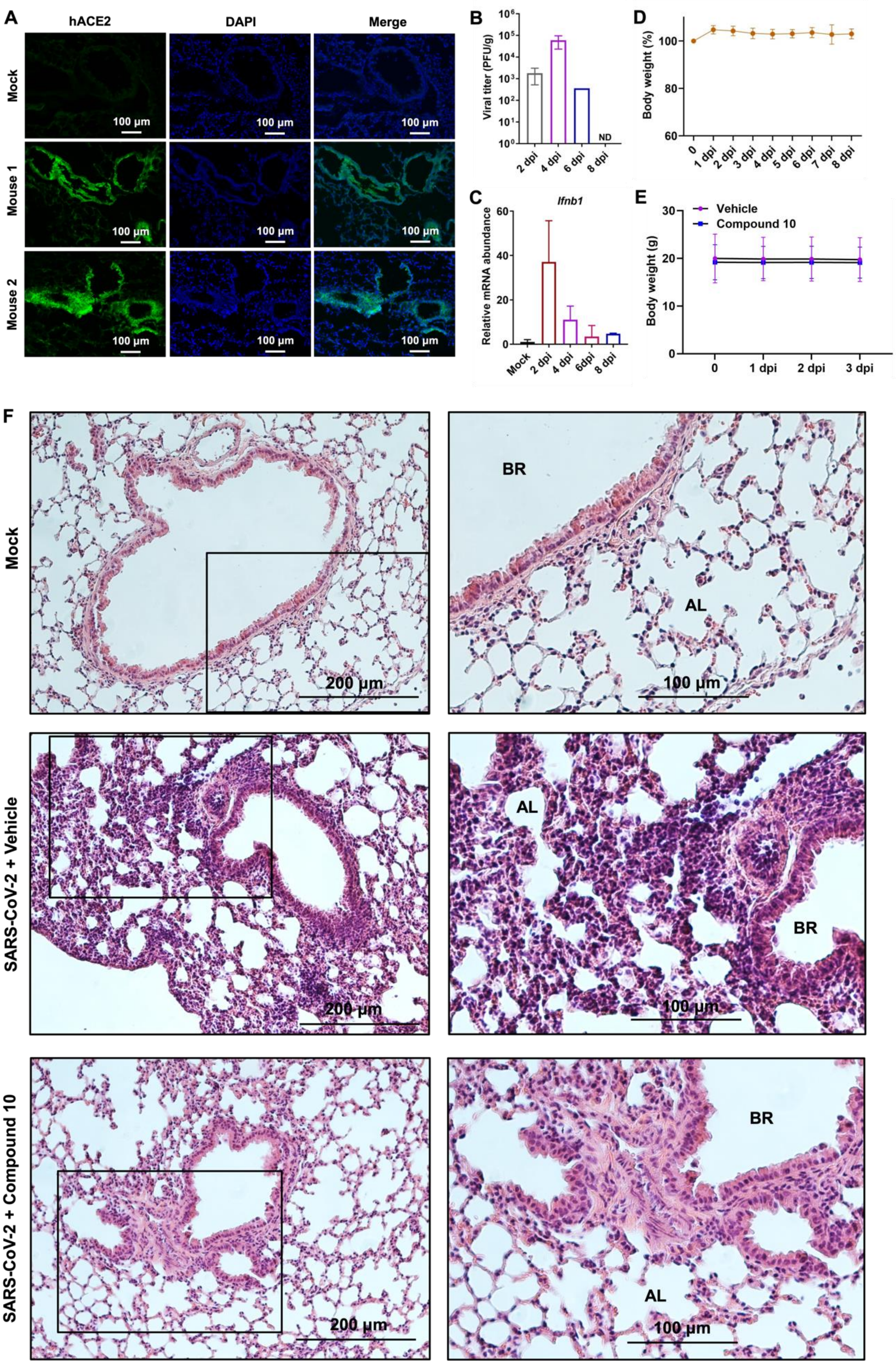

A
hACE2
DAPI
Merge
Mock
Mouse 1
Mouse 2
100 µm
B
Viral titer (PFU/g)
2 dpi
4 dpi
6 dpi
8 dpi
ND
D
Body weight (%)
0
1 dpi
2 dpi
3 dpi
4 dpi
5 dpi
6 dpi
7 dpi
8 dpi
C
Ifnb1
Relative mRNA abundance
Mock
2 dpi
4 dpi
6dpi
8 dpi
E
Vehicle
Compound 10
Body weight (g)
F
Mock
SARS-CoV-2 + Vehicle
SARS-CoV-2 + Compound 10
BR
AL
200 µm
100 µm

Fig S5 A CTPS1 inhibitor impedes SARS-CoV-2 replication and alleviates pathogenesis *in vivo*. (A) Immunofluorescence staining was performed on the frozen lung tissues from AAV-hACE2-transduced C57BL/6J mice with an antibody against ACE2 protein. (B and C) Viral titer and *Ifnb1* mRNA levels in lungs from AAV-hACE2-transduced C57BL/6J mice intranasally infected with 1.5 x 10^5 PFU of SARS-CoV-2 were determined by plaque assay or real-time PCR, respectively. (D) Body weigh of AAV-hACE2-transduced C57BL/6J mice intranasally infected with 1.5 x 10^5 PFU of SARS-CoV-2. (E) Body weight of vehicle or compound **10** treated K18-hACE2 transgenic mice infected with 1 x 10^4 PFU of SARS-CoV-2 from 1 to 3 dpi. (F) H&E staining of vehicle or compound **10** treated K18-hACE2 transgenic mice infected with 1 x 10^4 PFU of SARS-CoV-2 at 3 dpi. Data are presented as mean ± SD (B-E).

Table S1

Gene Target	Forward	Reverse
Real-time PCR primers for human genes		
IFNB1	CTTTCGAAGCCTTTGCTCTG	CAGGAGAGCAATTTGGAGGA
Mx1	GGTGGTGGTCCCCAGTAATG	ACCACGTCCACAACCTTGTCT
ISG15	GTGGACAAATGCGACGAACCCC	TCGAAGGTCAGCCAGAACAG
ISG56	TCTCAGAGGAGCCTGGCTAA	TGACATCTCAATTGCTCCAG
CCL5	CCTGCTGCTTTGCCTACATTGC	ACACACTTGGCGGTTCTTTCGG
CTPS1	AGCTTGGCAGAAGCTCTGTA	CCAACTGCATCCCTAAGCAC
β-actin	GTTGTCGACGACGAGCG	GCACAGAGCCTCGCCTT
Real-time PCR primers for mouse genes		
Ifnb1	CCCTATGGAGATGACGGAGA	CCCAGTGCTGGAGAAATTGT
Ifna4	GCAGAAGTCTGGAGAGCCCTC	TGAGATGCAGTGTTCTGGTCC
Isg15	TCCATGACGGTGTCAGAACT	GACCCAGACTGGAAAGGGTA
Cxcl10	CCTGCCCACGTGTTGAGAT	TGATGGTCTTAGATTCCGGATTC
Mx1	GTGGTAGTCCCCAGCAATGT	TGCTGACCTCTGCACTTGAC
Isg56	CAAGGCAGGTTTCTGAGGAG	GACCTGGTCACCATCAGCAT
Gapdh	GTTGTCTCCTGCGACTTC	GGTGGTCCAGGGTTTCTTA
Real-time PCR primers for SARS-CoV-2 genes		
Nsp1	ACACGTCCAACTCAGTTTGC	CGAGCATCCGAACGTTTGAT
E	ACTTCTTTTTCTTGCTTTCGTGGT	GCAGCAGTACGCACACAATC
N	GGGGAACTTCTCCTGCTAGAAT	GGGGAACTTCTCCTGCTAGAAT

S	GCTGGTGCTGCAGCTTATTA	AGGGTCAAGTGCACAGTCTA
sgRNA targeting sequences for human CTPS1		
CTPS1 sgRNA 1	GAATCATTGCCAGCAGTGT	
CTPS1 sgRNA 2	CCTTGACATCCGCCTCACCA	
